# Brain responses to repetition-based rule-learning do not exhibit sex differences: an aggregated analysis of infant fNIRS studies

**DOI:** 10.1038/s41598-024-53092-2

**Published:** 2024-01-31

**Authors:** Jessica Gemignani, Judit Gervain

**Affiliations:** 1 Department of Developmental and Social Psychology, University of Padua, Via Venezia 8, 35131 Padua, Italy; 2 Padova Neuroscience Center, University of Padua, Padua, Italy; 3 Integrative Neuroscience and Cognition Center, CNRS &, Université Paris Cité, Paris, France

**Keywords:** Language, Psychology

## Abstract

Studies have repeatedly shown sex differences in some areas of language development, typically with an advantage for female over male children. However, the tested samples are typically small and the effects do not always replicate. Here, we used a meta-analytic approach to address this issue in a larger sample, combining seven fNIRS studies on the neural correlates of repetition- and non-repetition-based rule learning in newborns and 6-month-old infants. The ability to extract structural regularities from the speech input is fundamental for language development, it is therefore highly relevant to understand whether this ability shows sex differences. The meta-analysis tested the effect of Sex, as well as of other moderators on infants’ hemodynamic responses to repetition-based (e.g. ABB: “mubaba”) and non-repetition-based (e.g. ABC: “mubage”) sequences in both anatomically and functionally defined regions of interests. Our analyses did not reveal any sex differences at birth or at 6 months, suggesting that the ability to encode these regularities is robust across sexes. Interestingly, the meta-analysis revealed other moderator effects. Thus in newborns, we found a greater involvement of the bilateral temporal areas compared to the frontal areas for both repetition and non-repetition sequences. Further, non-repetition sequences elicited greater responses in 6-month-olds than in newborns, especially in the bilateral frontal areas. When analyzing functional clusters of HbR timetraces, we found that a larger right-left asymmetry for newborn boys in brain responses compared to girls, which may be interpreted in terms of a larger right-left asymmetry in cerebral blood flow in boys than in girls early in life. We conclude that extracting repetition-based regularities from speech is a robust ability with a well-defined neural substrate present from birth and it does not exhibit sex differences.

## Introduction

For decades, research has been interested in exploring whether females and males possess different linguistic abilities. While the empirical evidence is unambiguous on the fact that some language-related disorders like autism, dyslexia and stuttering affect sexes differently^1–3^, with males consistently reported to be more affected than females, research conducted on typical samples has provided contrasting results^4^. Females have often been shown to perform better than males on a number of language measures. For instance, they possess better word comprehension skills, a larger vocabulary and produce longer and syntactically more complex sentences^5–8^. However, a number of conceptual and methodological issues qualify a simple “female advantage” interpretation.

First, differences in linguistic performance are likely to arise from a complex interplay of biological differences^9–12^ and environmental factors. In fact, prenatal and early postnatal hormones are known to have a crucial impact on the developing brain^3^ and have been hypothesized for a long time now to be contributing to a prenatal sexual differentiation in cognitive abilities^13^. Recent work also identified different patterns of functional connectivity between sexes while still in utero^14^. However, psychosocial variables are not less relevant. For instance, evidence suggests that the quantity of input speech from parents differs between sexes^15,16^, and that the family’s socio-economic status^17^ and the socio-emotional engagement also affect sexes differently^18^. In other words, with the complex and continuous interaction between biology and environment, it is challenging to measure to what extent each of these factors contributes to sex differences in linguistic performance, and certainly it cannot be straightforwardly clarified within single studies.

Second, and relatedly, the effect sizes reported for the “female advantage” in single studies are typically very small^4^, and when taken together with other predictors, sex accounts for only a minor share of the observed variance (e.g. 1–2% in^5^). Sex differences are, therefore, generally investigated by means of meta-analyses, which extend the sample sizes and thus the statistical power of any single study^19^.

Third, available findings are unclear about the developmental origins of sex differences in language. For instance, while Maccoby and Jacklin^20^ reported them to occur mostly after the age of 11 years, Bornstein et al.^16^ found them to be significant only until 6 years of age. Zambrana et al.^21^ reported girls to have better language comprehension abilities than boys at 18 months and to a lesser extent at 36 months of age, while Le Normand et al.^22^ found that girls produced more words than boys up to 3 years of age, but not after that. Importantly, studies often fail to distinguish between sex differences in language development and differences in overall maturational speed between sexes. Furthermore, age has often been analysed using different age ranges and age groups across different studies, making comparisons more difficult^6^ and in fact some authors suggested that, rather than using age ranges, studies on sex differences should focus on very specific and relevant time points^12^.

Fourth, in many studies, potential sex and gender differences are simply overlooked as they are not statistically tested for in any systematic way. This masks possible differences, limits the generalizability of findings and may be a particularly important hinderance to interventions, e.g. in language pathologies, if differences in how the two sexes respond to treatment remain unexplored. Various funders, journals and research consortia now, therefore, recommend or even require that the sex and gender dimension be taken into account in any research involving humans and animals (e.g. Sex and Gender Equity in Research guidelines^23^. Yet, in neuroimaging studies of language development, sex and gender differences have very rarely been explicitly tested.

For all these reasons, the study of sex differences in language abilities has so far yielded inconsistent results, especially in development. Neuroimaging studies carried out very early in life have the potential to meaningfully contribute to this research question while circumventing some of the above mentioned methodological challenges. By testing very young infants, i.e. those with none or very little experience with the environment, the contribution of socio-cultural factors can be reduced, thus affording the opportunity to get closer to the biological basis of any sex difference. Furthermore, by measuring brain function associated with language processing, rather than its behavioral reflections, which are very challenging to test in young infants, neuroimaging studies have the potential to provide a more sensitive measure of sex differences and their developmental trajectory.

The current work, therefore, addresses the question of early sex differences in language processing by conducting a meta-analysis of neuroimaging studies carried out with functional near-infrared spectroscopy (fNIRS) on newborn and 6-month-old infants with the aim of investigating whether one of the earliest language abilities, the extraction of linguistic regularities from speech, shows sex differences.

We have chosen to investigate this ability as it is a very basic mechanism that is in place already at birth and that has been found to be foundational for learning grammar and thus for language development. At birth, infants have been shown to be able to extract structural regularities from speech. In particular, they can learn and generalize repetition-based rules, i.e. the identity relation (A = A), which allows them to discriminate between artificial grammars with ABB (e.g. “mubaba”, “penana” etc.), AAB (e.g. “babamu”, “nanape” etc.) and ABA (e.g. “bamuba”, “napena” etc.) structures and distinguish them from random controls (ABC: “mubage”, “penaku” etc.;^24^). Repetition-based rule learning is considered a basic mechanism of language acquisition^25^ and has been investigated with both behavioral^26^ and neuroimaging methods, especially fNIRS^27^, but the question whether it shows any sex difference has never been investigated. Testing whether any sex difference may be observed for such an elemental mechanism, and at birth, would offer an important contribution to the understanding of sex differences in language processing and especially to the hypotheses about their biological basis. No currently available evidence points to the existence of sex difference in this ability, especially at such an early age, but as pointed out before, sex differences are typically not tested for in NIRS studies, and even when tested in a single study, they may not be detected due to small sample sizes and high individual variability in the data. This, however, may lead to undetected effects and less inclusive, potentially unequitable results, which may falsely generalize findings found predominantly for one sex/gender to the other or may fail to generalize effects tested only in one gender to both. Therefore, the availability of a highly coherent dataset of fNIRS studies with young infants, tested in very similar paradigms, offers a unique opportunity to test for potential sex differences.

fNIRS is a non-invasive technique for functional imaging of brain hemodynamics^28^. Relying on the different absorption spectra of oxygenated and deoxygenated hemoglobin (HbO and HbR, respectively) in the near-infrared region of the electromagnetic spectrum (650–900 nm), fNIRS measures the relative changes of oxygenation in the human brain at rest or in response to stimulation. As fNIRS is non-invasive, portable, silent and relatively robust with respect to motion artifacts, it is widely employed in developmental cognitive neuroscience research, and in particular in research on early speech perception and language development^29^.

**Figure 1:**
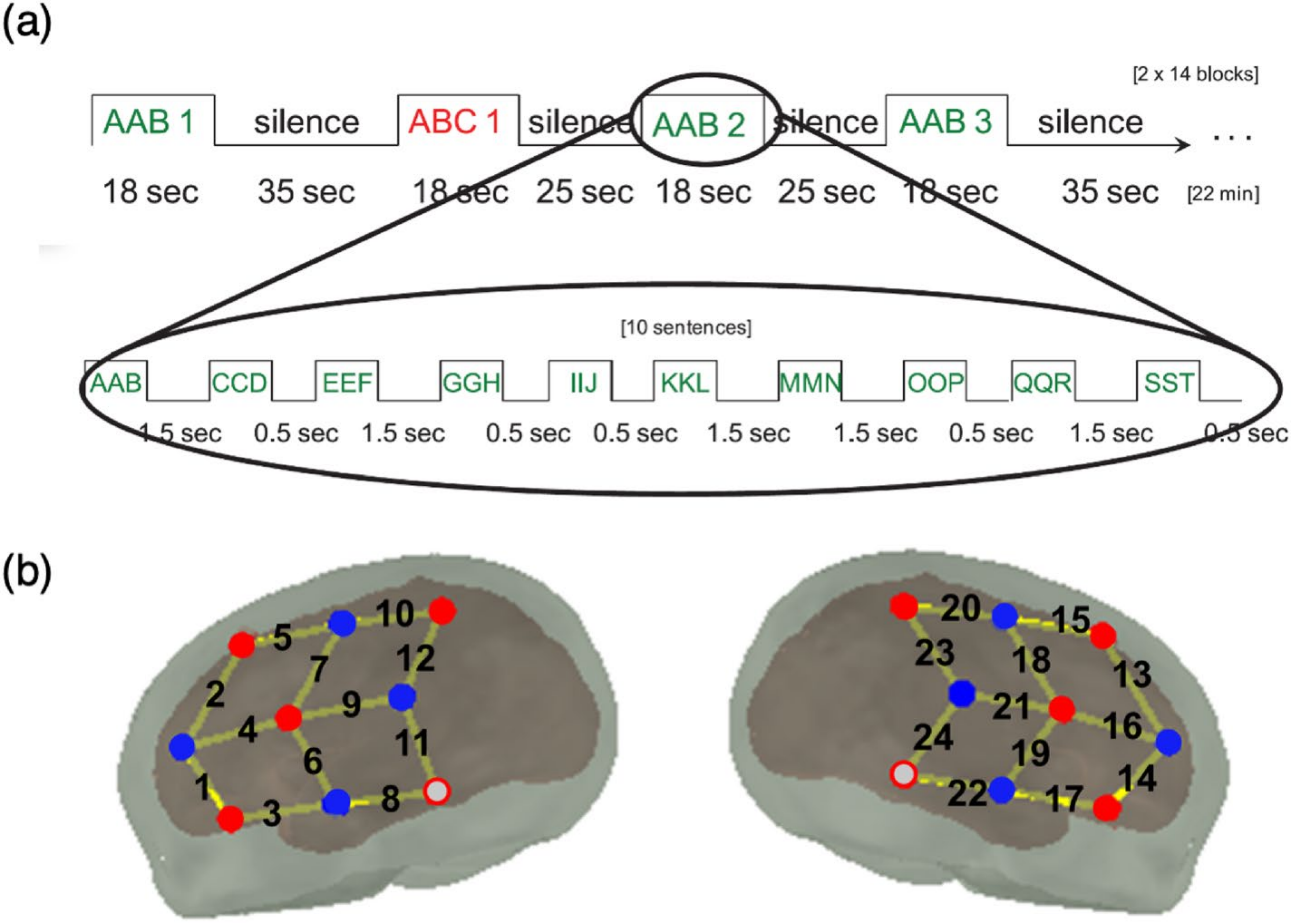
(**a**) A typical experimental design, with repetition-based- (AAB, in this specific example) and non-repetition-based regularities (ABC) being presented to infants in blocks (figure adapted from^24^) (**b**) The optode arrangement employed in the studies included 8 or 10 sources (red dots) and 8 detectors (blue dots), forming a total of 20 or 24 channels. Grey dots with a red outline indicate sources that were not present in studies 2 and 5. Anatomically relevant regions described in Sect. 2.2.3 are the bilateral frontal area (LH: channels 2, 5; RH: 13, 15) and the bilateral temporal area (LH: channels 3, 6; RH: channels 17, 19). The anatomical localization of the array is described in detail in^30^).

In the current work, we have gathered seven published and unpublished fNIRS studies, carried out on newborns and six-month-old infants in three laboratories, and explored whether patterns of neural responses underlying infants’ rule learning exhibited sex differences. The seven studies (Table 1) used very similar paradigms, whereby sequences generated by artificial grammars following repetition-based (e.g. ABB: “mubaba”, “penana” etc.; AAB: “babamu”, “nanape” etc.) and/or non-repetition-based (ABC: “mubage”, “penaku” etc.) regularities were presented in blocks to infants (Fig. 1a), while NIRS measures were obtained from the bilateral temporal, frontal and parietal areas covering the language network (Fig. 1b).

**Table 1:** Main characteristics of the studies included in the present meta-analysis.

Study	Location	Grammars	Trials per Grammar	Age (m)	fNIRS device	Wavelenghts (nm)	Sampling rate (Hz)	M	F
1. Bouchon^41^	Paris, France	AAB, ABC	14	0	NIRx	760, 850	10	9	15
2. Radulescu et al. (in preparation)	Paris, France	AAB, ABC	6	6	NIRx	760, 850	15.62	10	11
3. Bouchon et al.^42^	Paris, France	ABB, ABC	12	0	NIRx	760, 850	15.62	11	12
4. Berent et al.^43^	Paris, France	AA, AB	7	6	NIRx	760, 850	15.62	11	12
5. Berent et al. (in preparation)	Paris, France	AA, AB	7	6	NIRx	760, 850	15.62	8	7
6. Gervain et al.^24^	Vancouver, Canada	AAB, ABC	14	0 m	Hitachi	695, 830	10	13	9
7. Gervain et al.^44^	Trieste, Italy	ABB, ABC	14	0 m	Hitachi	695, 830	10	10	12
Total								72	78

All studies employed two auditory conditions, one containing adjacent repeated syllables, the other containing random non-repetead syllables; Trials refers to the number of trials administered for each condition.

Meta-analyses are especially valuable for this type of research questions, as they offer greater sample sizes and statistical power and are thus better able to detect small effects. They have been successfully conducted over behavioral data^31,32^, but very few, to date, have been conducted on developmental fNIRS data^33,34^, despite the pressing need in the developmental fNIRS community for efforts aimed at addressing issues of replicability^35^. To our knowledge this is the first meta-analysis to address sex differences in neural mechanisms during early human development, measured with fNIRS. We have gathered all the existing studies that addressed this issue and that possessed the relevant demographic information.

While the number of studies included in our analysis is not particularly large, it is now increasingly recognized in the literature that even small-scale meta-analyses, aggregating over as few as two studies, help gain significant insight by increasing sample sizes, variability covered and representativity^36^ In fact, several authors argue that, whenever a series of conceptually comparable studies is available, internal meta-analyses should be routinely carried out, as they allow moving away from focusing on p-values of individual studies and identifying potential moderators of variability^37–40^.

This latter point is especially relevant for our case, as sex differences in language development are small, and thus require large sample sizes to be statistically detectable. We are thus running this comprehensive analysis with the specific goal of increasing sample size and thus be able to find the effect of sex, if present.

In particular, our analysis includes a cohort of newborns (n = 91) that is much larger than the usual sample size of single studies^33^. The study of this developmental time point is particularly relevant as the impact of environmental factors immediately at birth is still smaller than later in development. We can thus more readily probe the biological basis of sex differences in language development. Secondly, including studies with six-month-old infants (n = 59) elucidates the developmental trajectory of putative sex differences underlying rule learning. Since rule learning is foundational for later language acquisition, the question whether its neural correlates show sex differences early in development is highly relevant for our understanding of differences found in later linguistic performance.

**Figure 2:**
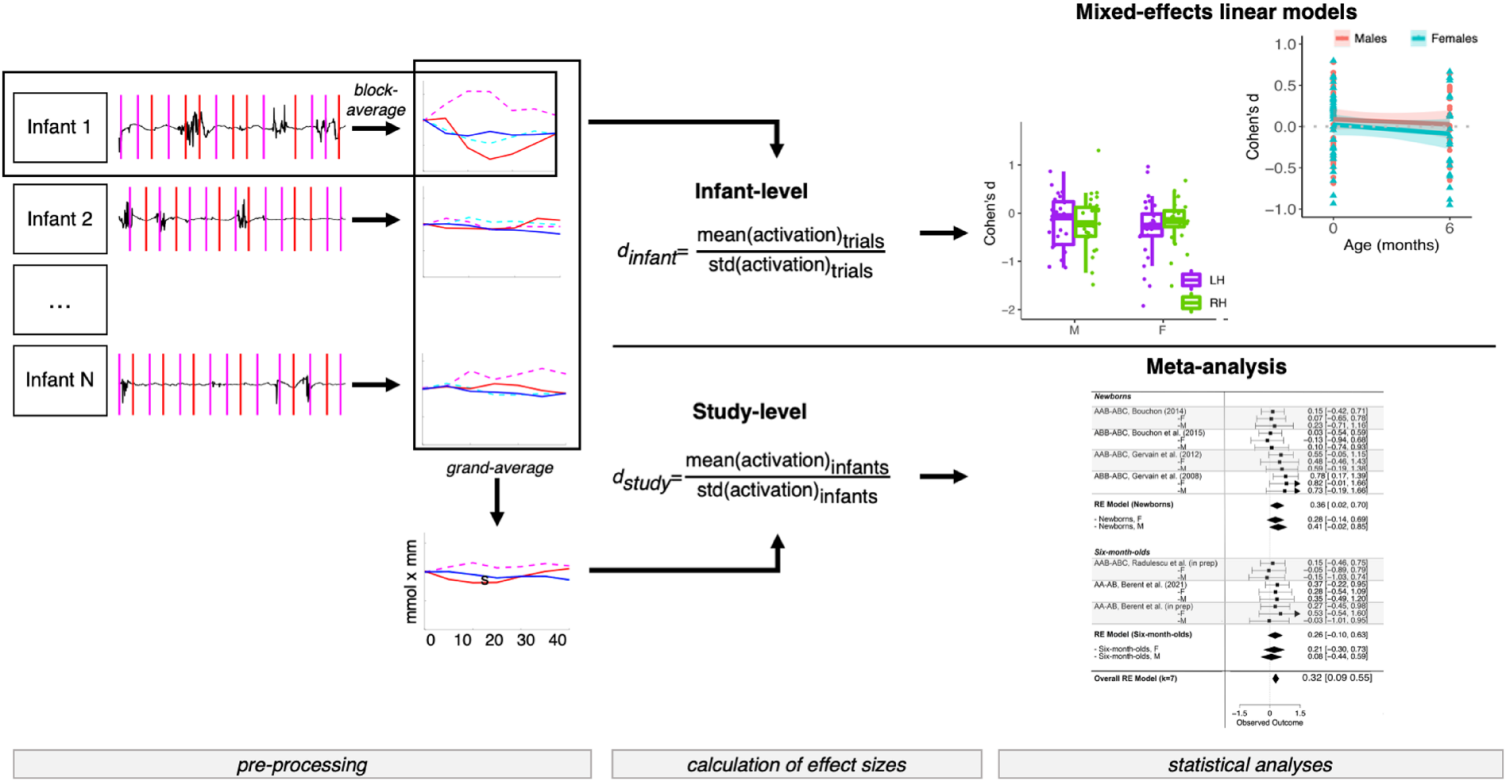
Schematic illustration of the methodology employed. (*Pre-processing*) Within each study, subject-wise block-averages are computed, as well as study-level grand-averages; in the plots, magenta and cyan indicate repetition trials (HbO and HbR, respectively), red and blue indicate non-repetition trials (HbO and HbR, respectively). (*Calculation of effect sizes*) After pre-processing data, for each study infant-level and study-level effect sizes are computed as described in Section “Calculation of effect sizes”, where Activation refers to the R vs. 0, N vs. 0 and R vs. N contrasts computed as the average of the HRF along its time course. (*Statistical analyses*) Infant-level effect sizes are analysed through mixed-effects linear models, investigating the effects of Sex, Age, Regions of Interest and Hemisphere; plots reported are examples of such analysis. Study-level effect sizes are analysed though meta-analytic methods, aimed at estimating effects within each subset of data and at investigating the moderating effects of Sex.

This paper takes two complementary methodological approaches. First, it employs state-of-the-art meta-analytic techniques to estimate the overall magnitude of the effect size of neural responses to two different linguistic rules, repetition- (“R”) and non-repetition-based (“N”) regularities, across studies, and compares them across sexes. Second, it statistically compares effect sizes across sexes using a linear mixed effects model. A schematic illustration of the methodology is provided in Fig. 2. The analyses concentrate on the effects of Sex, and within-subject variables inherent to the NIRS data such as Hemisphere or Region-of-Interest. We abstract away from other factors that the meta-analysis could potentially address, such as cross-lab variability, as they have been reported elsewhere^34^.

## Results

We performed analyses using two different and complementary approaches: a meta-analytical approach, carried out on study-level effect sizes and a mixed-effects modeling approach, carried out on infant-level effect sizes (Fig. 2). The meta-analytic framework estimates the variability of effect sizes across studies. It can be conducted even when only group-level averages, but not individual participant data, are available and when procedures or data types are not standardized. When trial-level data for each participant is available, it is also possible to compute individual effect sizes and perform a mixed-effects model, yielding a more sensitive measure of within-study variability. We thus calculated both study-level and infant-level effects sizes, both across and between sexes, and analysed the former with meta-analytic regression, and the latter with mixed-effects linear models.

### Meta-analysis

Using meta-analytic regression, we fitted intercept-only random-effects models to the study-level effects, in order to estimate the magnitude of the effects of interest within Sexes, Regions-of-interest (ROIs) and Ages. Regions-of-interest were chosen anatomically, covering the classical language network: the bilateral temporal areas (channels 3, 6 in the LH and 17, 19 in the RH, Fig. 1a, known to be responsible for auditory processing, and the bilateral frontal areas (channels 2, 5 in the LH and 13, 15 in the RH), responsible for the computation of structure and higher-order linguistic/sequential representations, following Gervain et al.^24,44^. In addition to estimating the magnitude of the effects, we analysed whether they showed any moderating effect of sex, for each region. Other variables that could potentially moderate the effect sizes, such as the position of the repetition or the different laboratories where the studies were carried out, have been found non-significant in modulating the variability of the rule learning effect in previous work^34^, therefore they will not be investigated in the current work.

#### Estimates of the effects

##### R vs. 0

**Figure 3:**
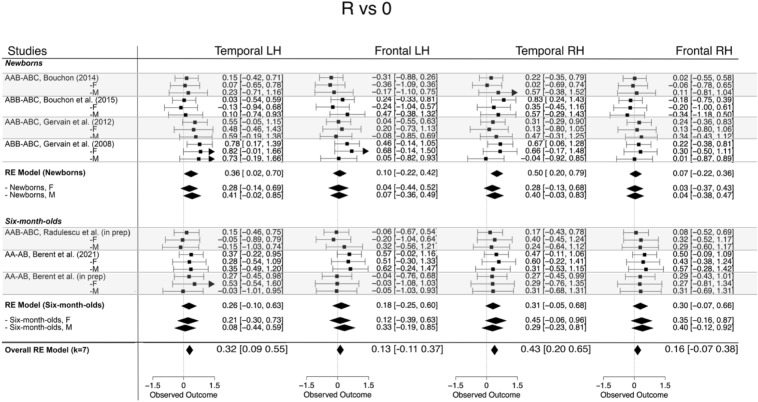
Forest plots of the effect sizes and corresponding confidence intervals obtained for responses elicited by repetition-based sequences compared to baseline. The bottom row, labelled as “Overall RE Model (k = 7)” reports the results of the meta-analysis carried out within each anatomical region across age groups and sexes. Summary ‘diamonds’ show the summary estimates of each group, based on the results of the model, with the center of the diamond corresponding to the estimate and the left/right edges indicating the confidence interval limits. The estimates for the two sexes are indicated for each study. The corresponding HbR forest plot is shown in Fig. S1.

The overall magnitude of the response to repetitions as compared to baseline across all regions of interest and age groups was 0.256 (95% CI [0.142, 0.370], *z* = 4.41, *p* < 0.001). Across the different ROIs, it was 0.132 (95% CI [− 0.117 0.371], *z* = 1.08, *p* ns) in the left frontal, 0.320 (95% CI [0.089, 0.546], *z* = 2.72, *p* < 0.01) in the left temporal, 0.155 (95% CI [− 0.071, 0.382], *z* = 1.34, *p* ns) in the right frontal and 0.425 (95% CI [0.195, 0.654], *z* = 3.62, *p* < 0.001) in the right temporal areas. When comparing the two age groups, the estimates were larger for newborns than for the 6-month-olds in the bilateral temporal areas. The corresponding forest plots are shown in Fig. 3. The full details of the results for the overall and subgroup analyses can be found in the Supplementary Material in Table S1.

##### N vs. 0

**Figure 4:**
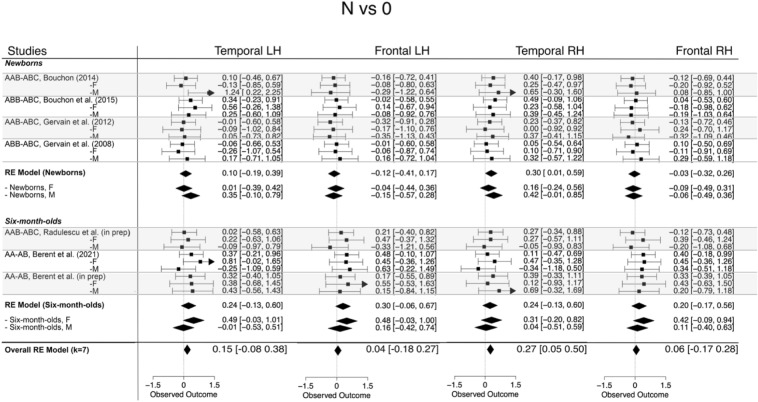
Forest plots of effect sizes and corresponding confidence intervals obtained for responses elicited by non-repetition-based sequences compared to baseline. The corresponding HbR forest plot is shown in Fig. S2.

The overall magnitude of the response to non-repetition sequences was 0.131 (95% CI [0.018, 0.244], *z* = 2.26, *p* < 0.05). Across the different ROIs, it was 0.043 (95% CI [− 0.18, 0.27], *z* = 0.37, *p* ns) in the left frontal, 0.151 (95% CI  [− 0.07, 0.38], *z* = 1.3, *p* ns) in the left temporal, 0.057 (95% CI [− 0.17 0.28], *z* = 0.49, *p* ns) in the right frontal, and 0.27 (95% CI [0.05, 0.50], *z* = 2.36, *p* < 0.05) in the right temporal areas. When comparing the two age groups, the estimates were larger for the 6-month-olds than for newborns in the bilateral frontal regions. Figure 4 shows forest plots for this effect. Full details are reported in the Supplementary Material (Table S1).

##### R vs. N

**Figure 5:**
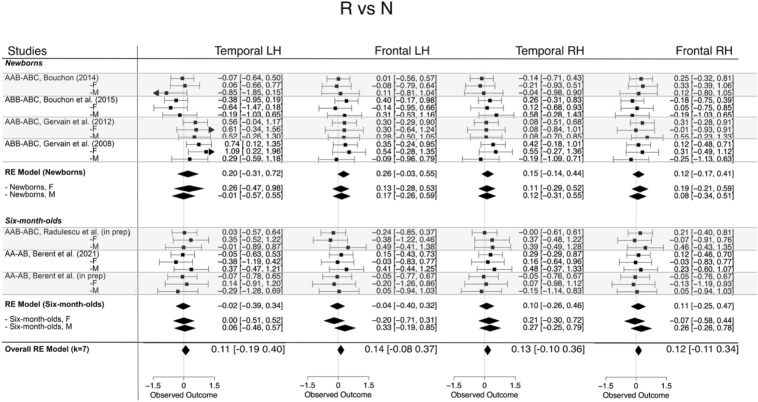
Forest plots of effect sizes and corresponding confidence intervals obtained for responses elicited by repetition-based sequences compared to non-repetition-based sequences. The corresponding HbR forest plot is shown in Fig. S3.

The overall magnitude of the effect was 0.122 ([0.009, 0.236], *z* = 2.122, *p* < 0.05) with a positive effect indicating a greater response to repetition sequences. Across the different ROIs, it was 0.144 (95% CI [− 0.08 0.37], *z* = 1.25, *p* ns) in the left frontal, 0.106 (95% CI [− 0.19, 0.40], *z* = 0.70, *p* ns) in the left temporal, 0.116 (95% CI [− 0.11 0.34], *z* = 0.99, *p* ns) in the right frontal and 0.131 (95% CI [− 0.1 0.35], *z* = 1.13, *p* ns) in the right temporal areas. When comparing the two age groups, the effect was larger in newborns than in 6-month-olds. Figure 5 shows the forest plots for this effect. Full details are reported in the Supplementary Material (Table S1).

#### Moderator analysis: the effect of sex

To answer our research question, we assessed whether female and male infants’ responses to repetition- and non-repetition sequences was significantly different. For each contrast, ROI and age group, meta-regression models were fitted that included sex as a moderator. In neither of the models was the effect of sex found to be statistically significant at *α* = 0.05. Figures 3, 4 and 5 show the effects broken down by sex for each study to illustrate that effect sizes were very similar for male and female infants. Full details can be found in the Supplementary Material.

### Linear mixed effects model

To better assess whether Sex, Region-of-Interest and Hemisphere have statistically significant effects, we also ran linear mixed effects models for the three comparisons (R vs. 0, N vs. 0, R vs. N) and the two age groups separately, using infant-level effect sizes (we also ran the analyses for the three comparisons with Age as a factor in addition to Sex, ROI and Hemisphere. The results are qualitatively very similar to the ones reported here, and are shown in the Supplementary Material). We built all possible models by incrementally adding the factors Sex, ROI and Hemisphere and their interactions to the model as fixed effects. The model with the best fit to the data, i.e. achieving the lowest AIC, was retained as the final model for each analysis. All tested models with their respective AIC are listed in the Supplementary Material.

Moreover, in addition to using the anatomically-defined ROIs described above, since no a priori hypotheses were available as to whether sex differences may be observed in different anatomical regions of the brain or in hemodynamic response patterns, we also employed functionally-defined ROIs. To do this, we carried out a data-driven functional localization analysis in order to identify ROIs that significantly activate for repetition and non-repetition sequences in each study. These functional ROIs were identified using cluster-based permutation tests involving t-tests^45^ over oxygenated hemoglobin (HbO) concentrations, separately for all three contrasts (R vs. 0, N vs. 0, R vs. N).

#### Anatomically derived regions

**Figure 6:**
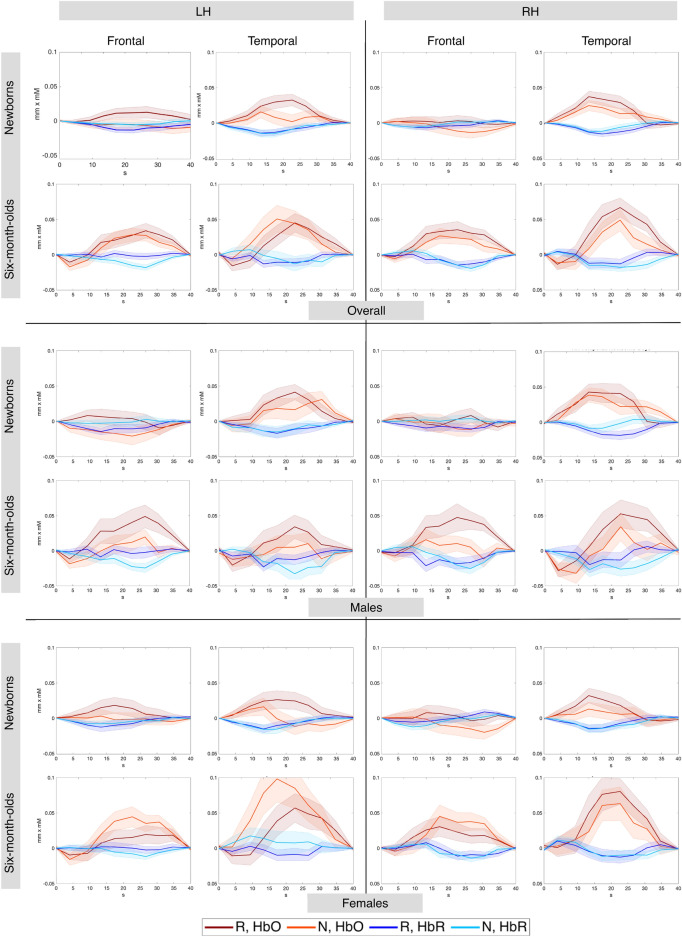
Grand average hemodynamic responses to repetition- and non-repetition sequences across all studies in the four anatomically defined regions of interest by age. Top panel shows the overall average, the middle and bottom panels show males and females, respectively.

Figure 6 shows grand average responses for repetition- and non-repetition sequences.

##### Oxygenated hemoglobin (HbO)

**Figure 7:**
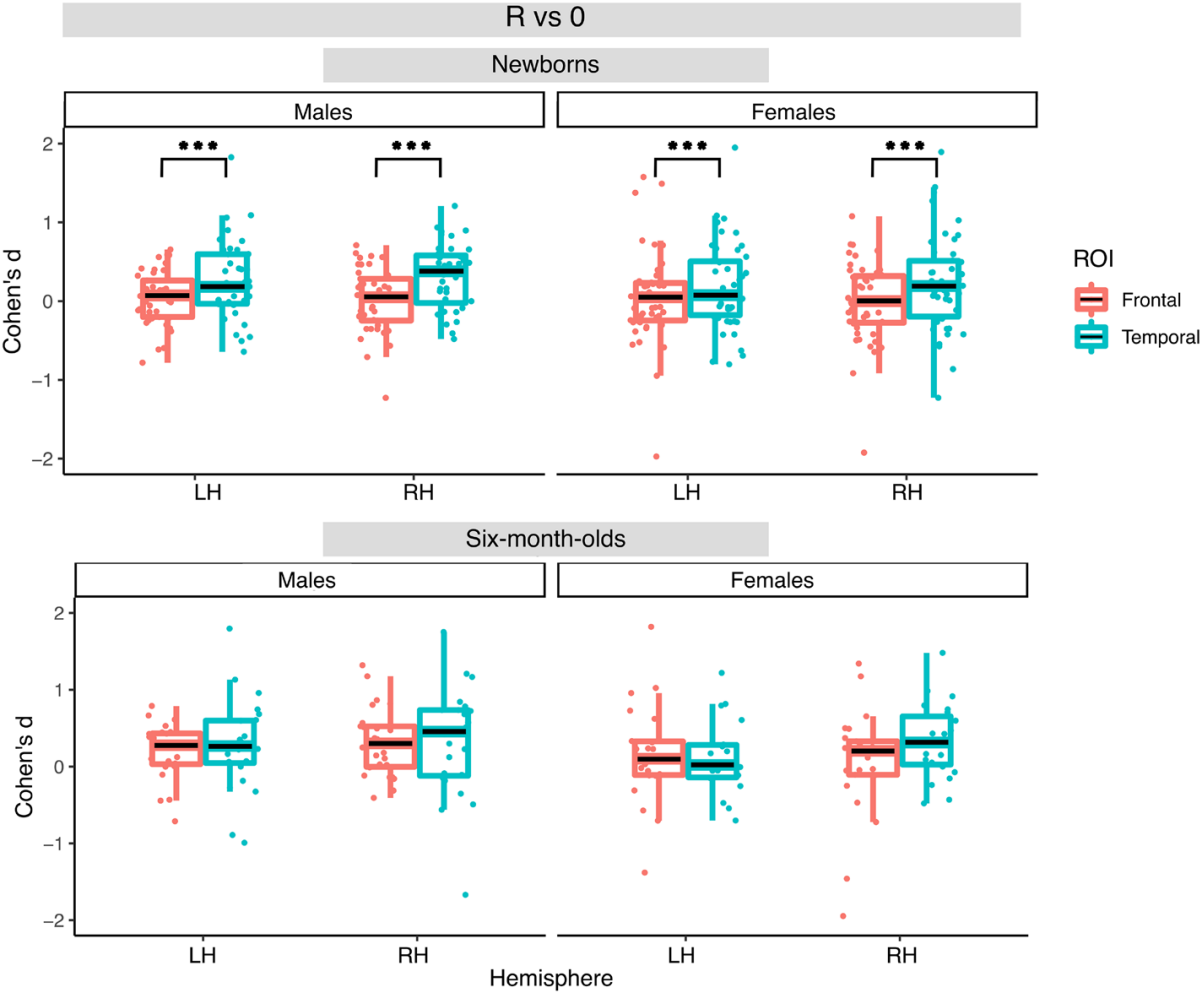
Box plots of infant-level effect sizes as a function of age, anatomically defined ROIs and hemisphere for responses elicited by repetition-based sequences compared to baseline. Boxplots display the median value of the distribution, its first and third quartiles (hinges) and whiskers extend to 1.5 times the interquartile range from each hinge.

**Figure 8:**
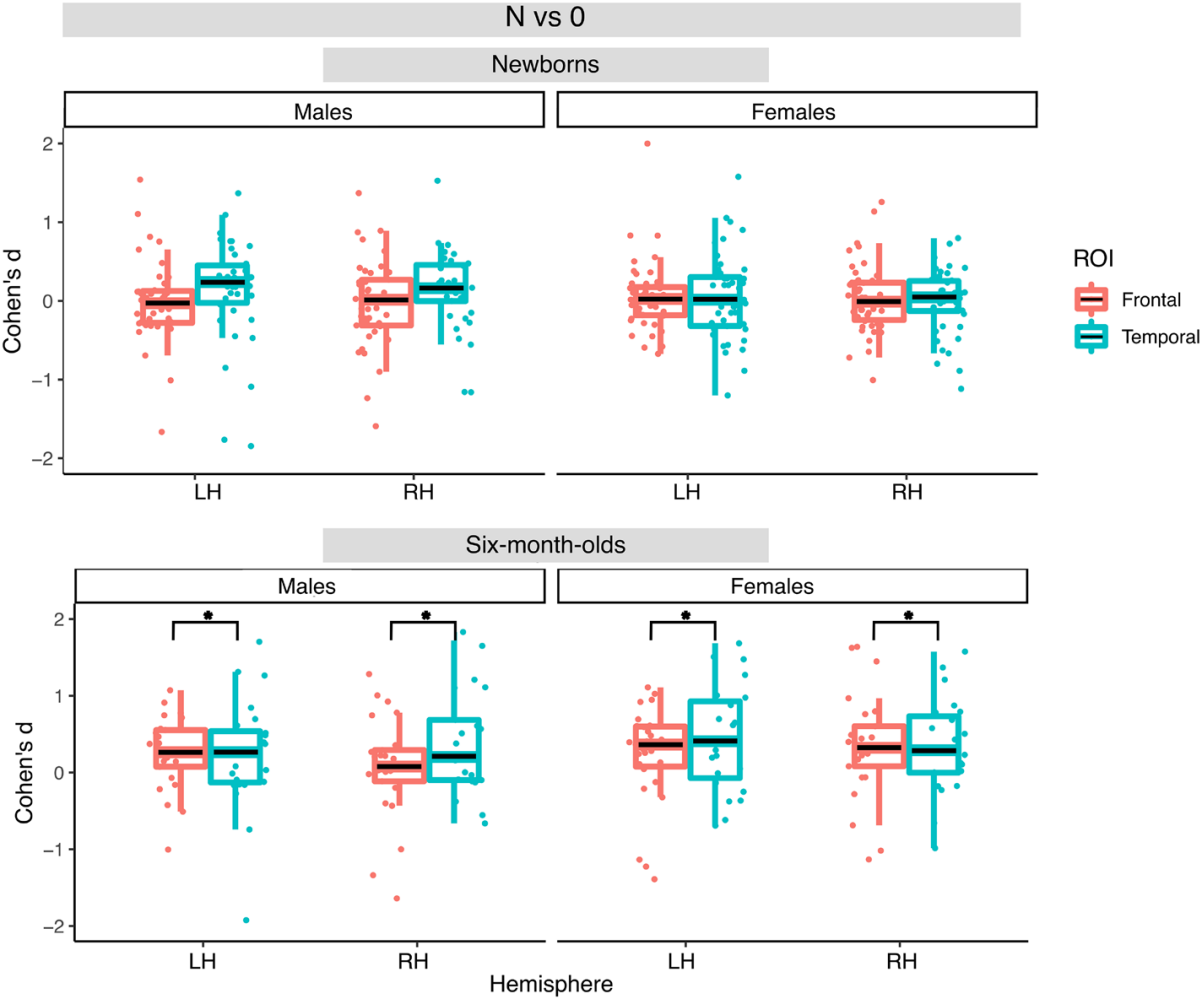
Box plots of infant-level effect sizes as a function of age, anatomically defined ROIs and hemisphere for responses elicited by non-repetition-based sequences compared to baseline.

**Figure 9:**
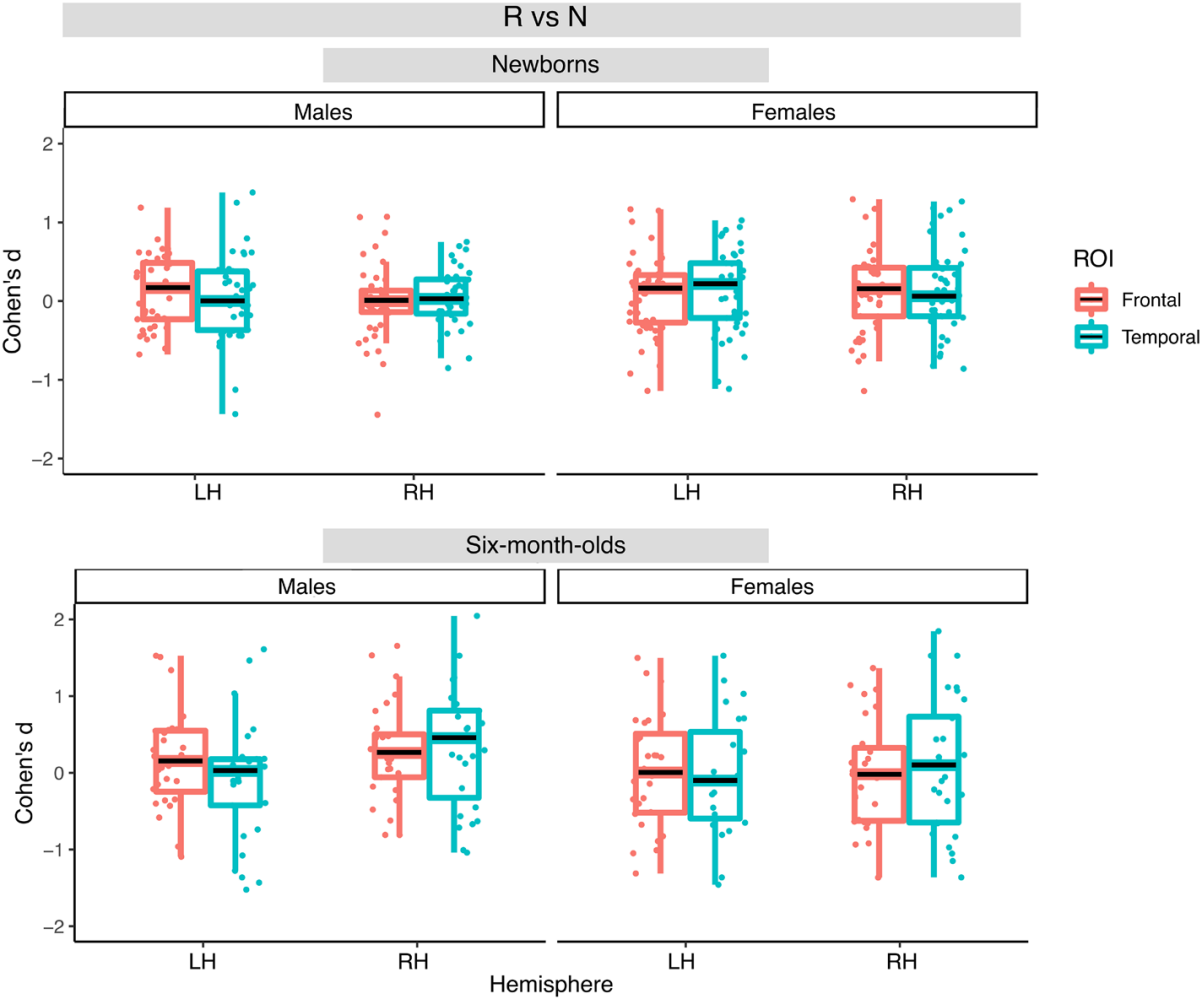
Box plots of infant-level effect sizes as a function of age, anatomically defined ROIs and hemisphere for responses elicited by repetition-based sequences compared to non-repetition-based sequences.

In newborns for the R vs. 0 contrast, the best fitting model included the fixed effect of ROI. This model yielded a significant main effect of ROI (F(1, 249) = 13.18, *p* < 0.001) with greater activation in the temporal ROIs than in the frontal ones. For the N vs. 0 and R vs N contrasts, the best fitting models also included the fixed effect of ROI, but did not yield significant effects. Figures 7, 8 and 9 illustrate distributions of the effects for the three contrasts, respectively.

In the 6-month-olds for the R vs. 0 contrast, the best fitting model included the fixed effect of Hemisphere, but did not yield any significant effect. For the N vs. 0 contrast, the best fitting model included the main effect of ROI, and yielded a significant effect of ROI (F(1, 135) = 4.90, *p* < 0.05) due to greater activation in the temporal than the frontal areas. No significant effects were found for the R vs. N contrast. Figures 7, 8 and 9 illustrate distributions of the effects for the three contrasts, respectively.

Importantly, no significant effect of Sex was found in any of the models.

##### Deoxygenated hemoglobin (HbR)

Similar analyses on HbR yielded no significant results (Figs. S4, S5 and S6).

#### Functionally derived regions of interest

**Figure 10:**
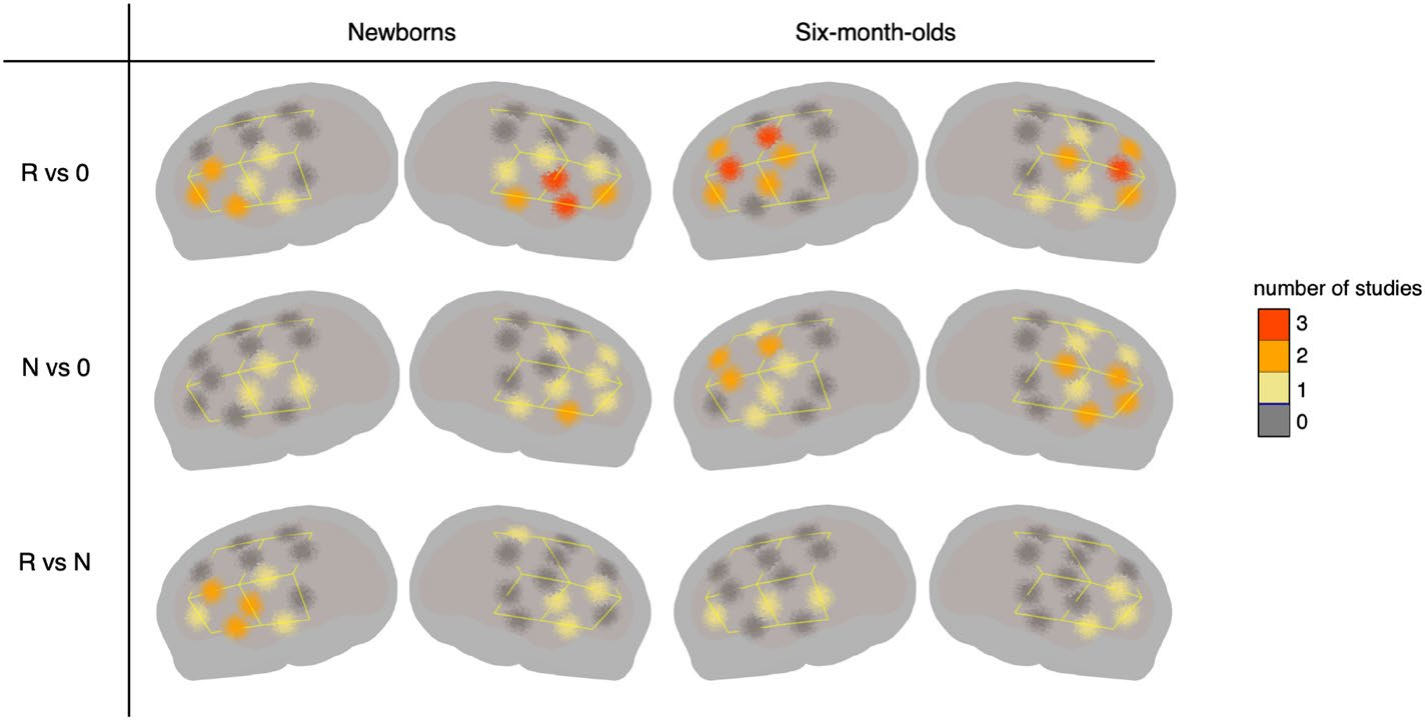
Results of the functional localization analysis: each channel is color coded to represent the number of studies in which it was found to show significant activation, for each of the three contrasts. Results of the analysis from each single study are listed in Table 2.

The functional localization analysis was used to identify one ROI per hemisphere for each study. Table 2 and Fig. 10 summarize the obtained ROIs (when the analysis identified no significant cluster, the average across all channels of that hemisphere was used in the statistical analyses). Effect sizes computed on these functional ROIs were analysed with linear mixed effects models similarly to anatomically defined ROIs.

**Table 2:** The channels constituting significant clusters in each study and each contrast (R vs. 0, N vs. 0, R vs. N).

Study	Stimuli (age)	R vs 0		N vs 0		R vs N	
		LH	RH	LH	RH	LH	RH
1	AAB-ABC (newborns)	*–*	19	*–*	*–*	*–*	*–*
2	AAB-ABC (6 m)	1,4,6,7,9	13,16,21	2,4,7	14, 16, 17	*–*	*–*
3	ABB-ABC (newborns)	1, 4	14, 17, 19, 22, 24	6, 9, 11	17	*–*	20
4	AA-AB (6 m)	1, 2,4,6, 7	14, 16, 17, 19, 21, 22	2, 4, 5, 7, 9	13, 15, 16, 18, 21	1, 6, 11	14, 16, 17
5	AA-AB (6 m)	2, 4, 7, 9	13, 14, 16, 18	3, 6	14,17, 19, 21	*–*	*–*
6	AAB-ABC (newborns)	3	17, 22	*–*	17,22	3, 4, 6, 8	*–*
7	ABB–ABC (newborns)	1, 3, 4, 6, 8, 9	14, 16, 17, 19, 21	*–*	13, 14, 16, 18, 19	1, 3, 4, 6, 9	16, 17, 19

*HbO*. In newborns, the best fitting model for the R vs. 0 contrast included only a fixed effect of Hemisphere, and it yielded no significant effects. For both N vs. 0 and R vs. N, the best fitting models included the fixed effects of Sex, but did not yield any significant effect.

**Figure 11:**
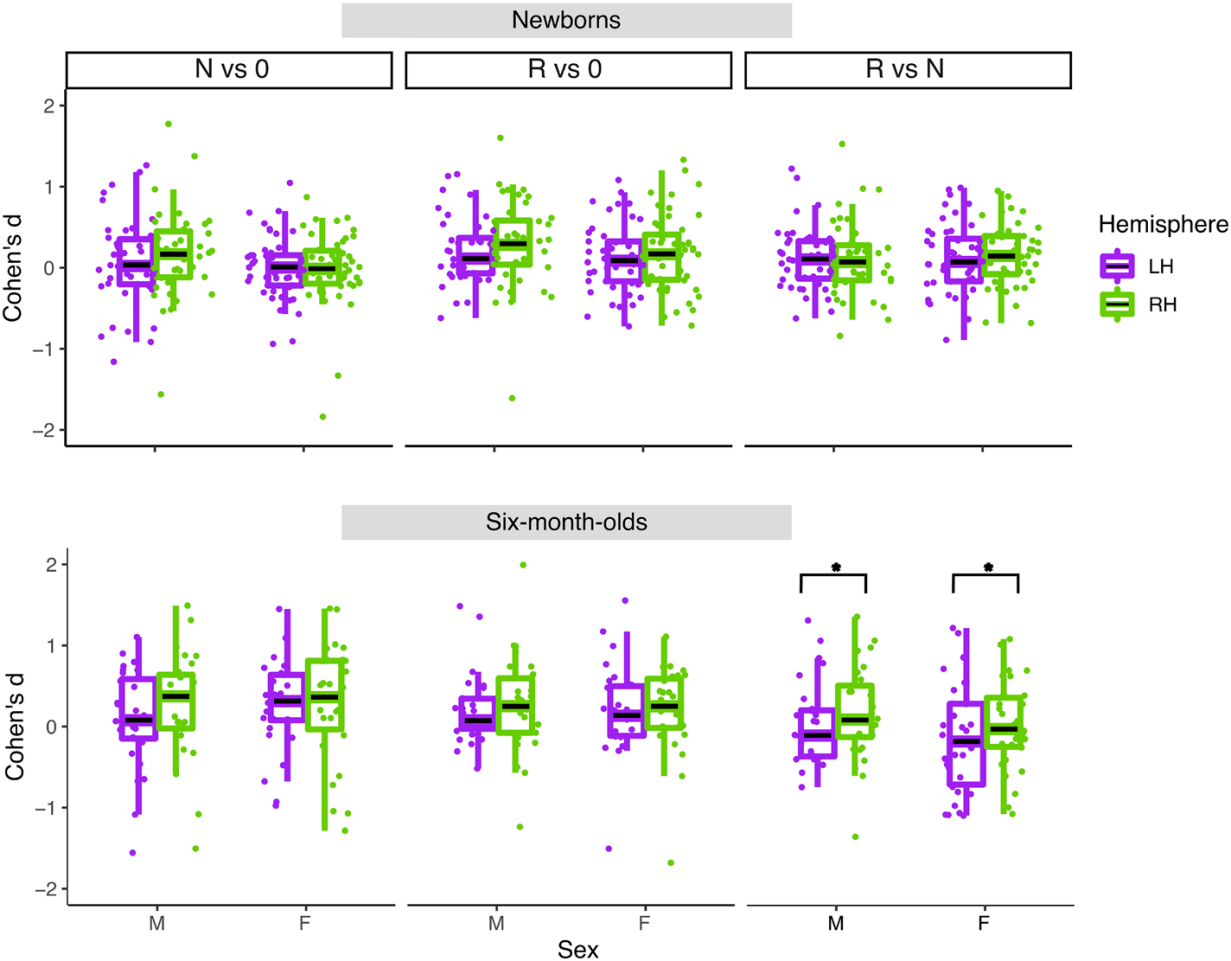
Box plots of infant-level effect sizes for the functionally defined ROIs in each hemisphere for the three contrasts in newborns (top panel) and 6-month-olds (bottom panel).

For 6-month-olds, the best fitting models for the R vs. 0 and N vs. 0 comparisons included Sex as a fixed factor, but did not yield any significant effect. The best fitting model for the R vs. N comparison included a fixed factor for Hemisphere, and it yielded a significant main effect (1, 46) = 6.42, *p* < 0.05), carried by a greater involvement of the RH compared to the LH. Figure 11 illustrates the distributions of the effects for the three contrasts in the two age groups.

##### HbR

In newborns, the best fitting model for the R vs. N contrast included fixed factors for Sex and Hemisphere as well as their interaction. The interaction was found to be significant (F(1, 83) = 4.61, *p* < 0.05), carried by a greater, i.e. more negative, effect in males in the RH than in females (estimate Males-Females = − 0.19, *p* < 0.05). No other significant effects were found in any of the other HbR analyses (Fig. S8).

**Figure 12:**
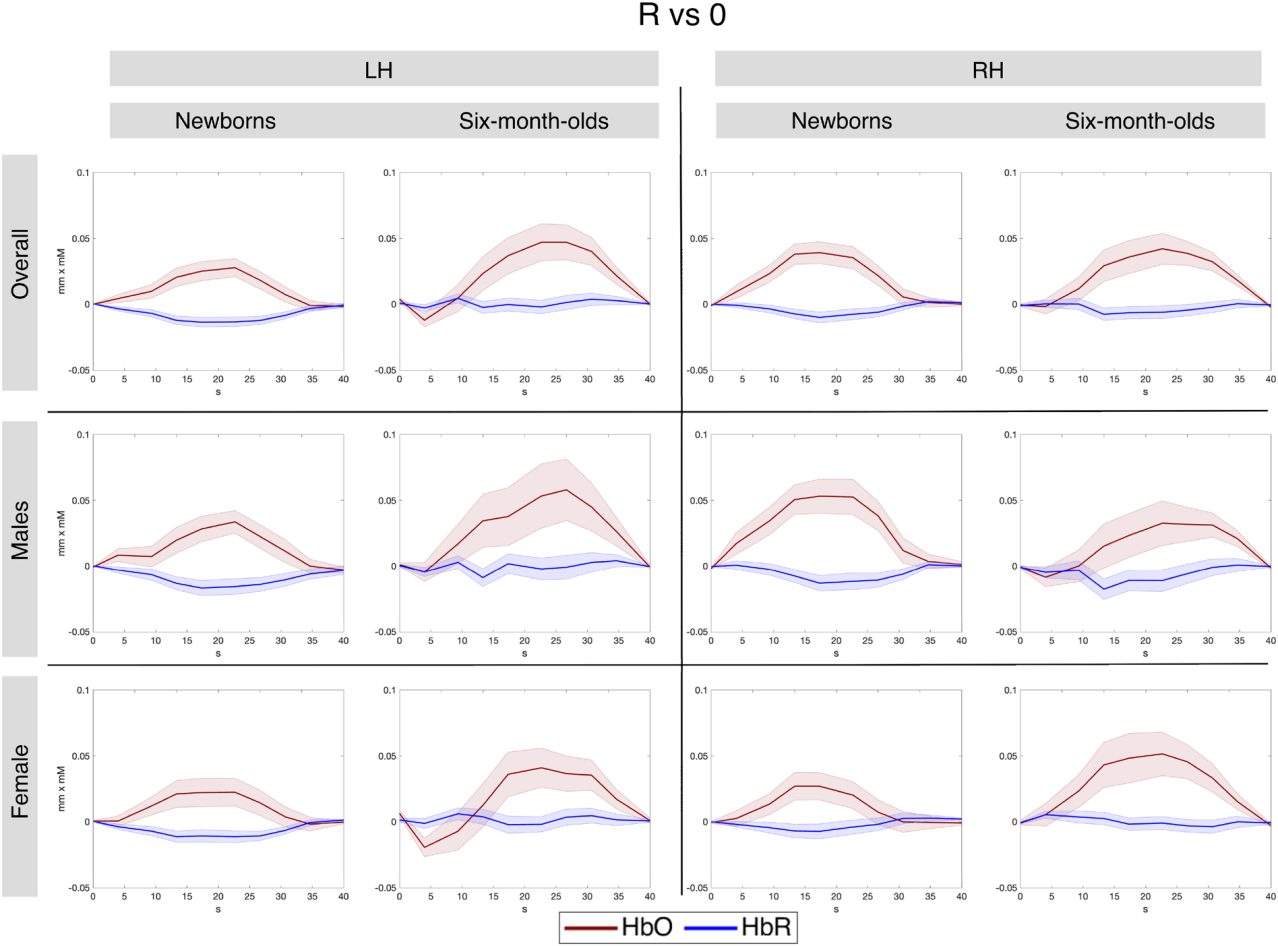
Grand average hemodynamic responses across all studies in the functionally defined regions of interest for the R vs. 0 contrast. Overall mean in the topmost panel, males in the middle and females in the bottom panel.

**Figure 13:**
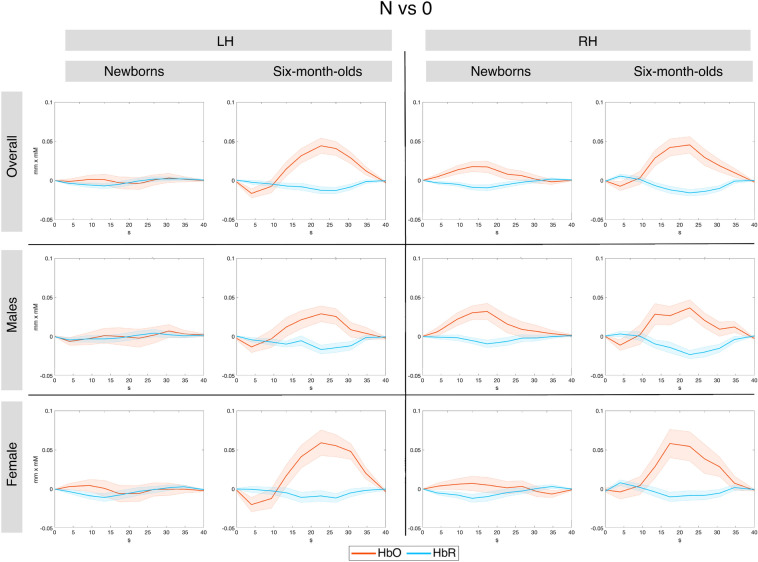
Grand average hemodynamic responses across all studies in the functionally defined regions of interest, for the N vs. 0 contrast.

**Figure 14:**
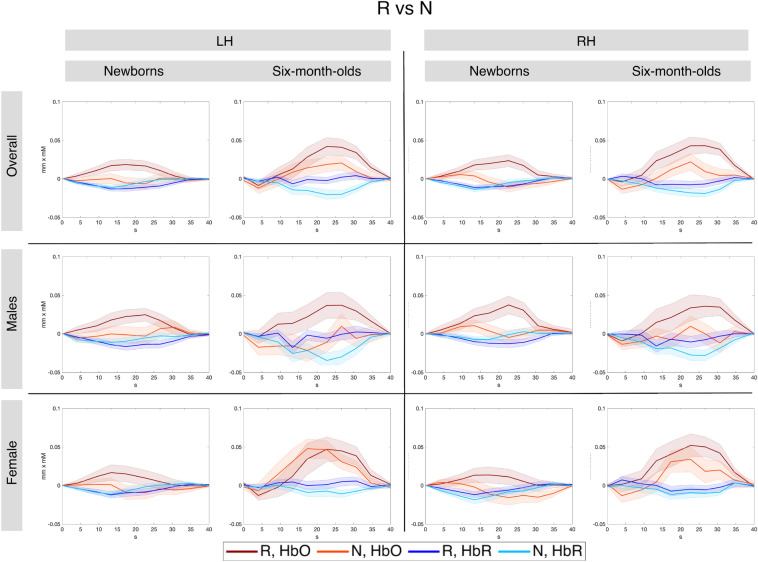
Grand-averages of hemodynamic responses to repetition- and non-repetition sequences, across all studies, within the functionally-defined regions of interest, for the R > N statistical comparison.

Figures 12, 13 and 14 show the grand averages of hemodynamic responses for the three contrasts (R vs 0, N vs 0 and R vs N, respectively) in the functionally-derived regions of interest.

## Discussion

The current study contributes to testing whether hitherto unexplored sex differences exist in early language abilities through a meta-analysis of fNIRS studies with newborns and six-month-olds, focusing on the ability to extract repetition- and non-repetition-based regularities from speech. Specifically, we tested whether brain responses to repetition and non-repetition regularities measured with fNIRS differed between males and females at birth and at 6 months of age. We investigated 7 fNIRS studies with very similar experimental designs, stimuli and setups. We tested sex differences using both a meta-analytic approach as well as linear mixed effects models. NIRS data was sampled from both anatomically and functionally defined regions of interest. In particular, we carried out separate analyses for three contrasts of interest, R vs 0 (repetition-based sequences against baseline), N vs 0 (non-repetition-based sequences against baseline) and R vs N (repetition-based- against non-repetition-based sequences) because they reflect three different brain mechanisms, conceptually independent of one another. The R vs 0 contrast is a test for the ability of repetition sequences to elicit a significant brain response with respect to the zero baseline. Similarly, the N vs 0 contrast tests the ability of non-repetition- or diversity-based, sequences to produce a significant brain response. These two mechanisms, repetition- and diversity-based rule learning, build on different cognitive mechanisms, the former is thought to be in place already at birth, while the latter is thought to develop at a later stage^27^, and hold different roles for subsequent language acquisition. Infants’ ability to learn repetition-based rules is instrumental for learning abstract patterns involved in grammar, while their ability to learn diversity-based structures has been related to the beginning of word learning, an ability that starts at around 6 months of age. The R vs N comparison, by contrast, reflects whether and how the infant brain is able to detect differences between two types of sequences.

We observed no sex differences in any of the analyses when comparing repetition-based sequences to baseline or to non-repetition sequences in newborns or in 6-month-olds.

The only result involving a sex difference in our analyses was found for hemispheric lateralization. We detected a larger right-left asymmetry for the differential response to repetition and non-repetition sequences in boys than in girls at birth. A closer look at the average effect sizes reveals that, qualitatively, this right-left asymmetry occurs for both types of sequences, even if it is not statistically significant. This suggests that this may be a general difference of hemispheric asymmetry between boys and girls, and not so much a language-specific result. Indeed, male infants have been reported to have a larger right-left asymmetry in cerebral blood flow than female infants at birth^46^. In addition to the effect sizes over which the statistics were conducted, the hemodynamic responses themselves also support this physiological interpretation (Figs. 12, 13). Moreover, this result is found uniquely for HbR, which is known to be a less reliable measure than HbO in infants^47,48^.

Overall, our results suggest that there are no sex differences early in life in the infant brain’s ability to extract basic structural regularities from the speech input. While language is an area where sex differences have been reported in behavioral studies, they have not been investigated systematically in the neural correlates of speech perception and language processing abilities. Our study is the first to show, on a large sample of newborns and 6-month-old infants, that such differences cannot be observed for the ability of extracting structural regularities from speech based on repetitions. Considering how fundamental this ability is for learning grammar, since reduplication is a productive morphosyntactic process in the majority of the world’s languages^49^, finding a robust ability that is uniform across the two sexes is not unexpected and may serve as a solid foundation for language development. Whether sex and gender differences arise in this ability later in development or whether this is an area of language where such differences never emerge remains an open question.

While not observing sex differences in rule-learning abilities, we obtained other interesting effects. In newborns, we found that the temporal region was more strongly activated by repetition sequences than the frontal area in both hemispheres (Figs. 6, 7), consistently with findings from single studies^24,44^ and with the overall maturational patterns of the newborn brain^50^. The meta-analytic findings confirm that bilateral temporal areas in the newborn brain exhibit large responses to both initial repetitions (AAB) and final repetitions (ABB), thus distinguishing them from random sequences, and in fact, no hemispheric lateralization was found for the differential response to repetition vs. non-repetition sequences (R vs N) either in anatomically–(Figs. 6, 9), or functionally-defined ROIs (Figs. 11, 14).

At 6 months, responses to the non-repetition sequences increased in the bilateral frontal area as compared to birth, although effect sizes remained greater in the bilateral temporal areas, as in newborns (Figs. 6, 8). The fact that the infant brain starts to encode non-repetition-based sequences by 6 months of age could indicate infants’ emerging ability, at this age, to encode and represent *diversity* in linguistic stimuli, as suggested by de la Cruz-Pavía and Gervain^27^. This ability is in line with and would support the beginning of word learning and grammar acquisition, which start emerging at this age.

Finally, we notice an interesting trend in the involvement of the frontal areas between birth and 6 months. In particular, the meta-analytic effect size is the lowest for repetition sequences in the left frontal region at 6 months (*d*_*R*>*0, 6-month*_ = 0.18), while in the right frontal areas it grows between birth and six months from 0.07 to 0.30. Consistently, functionally defined regions also showed a greater involvement of the RH than the LH at 6 months, in particular this asymmetry was statistically significant for the differential response to repetitions vs. non-repetitions (Figs. 11, 14). This asymmetry can either be physiological, produced by the faster maturation of the right hemisphere in early childhood^50–52^, or alternatively, could suggest an increased functional involvement of the right frontal cortex in processing repetitions. Further research will be needed to definitively tease apart the two explanations, but previous work already found the right frontal area to play a role in early speech processing. For instance, Dehaene-Lambertz et al.^53^ found the right prefrontal cortex to be significantly activated during the processing of native speech in awake 3-month-old infants but not in sleeping infants, a result that the authors suggested to be correlated with attention and memory retrieval mechanisms.

The current study was limited by the number of experiments in which demographic information about sex was available. It has to be noted, however, that the estimates of the effects, for each different comparison and age group, overlap fully with the estimates computed in a larger meta-analysis on rule learning in infants^34^, confirming that the sample analyzed in this study is representative of the larger one.

Importantly, our study did not find relevant differences between boys and girls in processing repetition-based regularities. This is not to imply that other aspects of language acquisition do not exhibit such differences. Since rule learning is only one of the bricks that build infants’ linguistic knowledge, differences may be present in other processes. As later language abilities have often been shown to significantly differ between sexes, studies targeting mechanisms other than rule learning will be required to assess sex differences in other domains.

## Conclusions

Brain responses underlying repetition-based rule learning, measured with fNIRS do not exhibit sex differences at birth or at six months of age, suggesting that rule learning may be a robust and foundational ability subserving the acquisition of grammar. However, we found a larger right-left asymmetry in activation for newborn boys compared to girls, which we attribute to the well-documented larger right-left asymmetry in cerebral blood flow in boys than in girls early in life. Additionally, in newborns, we found a greater involvement of the bilateral temporal areas compared to the frontal area for both repetition and non-repetition-based sequences. We also found that non-repetition sequences elicited greater responses in 6-month-olds than in newborns, especially in the bilateral frontal areas. As rule learning is foundational for language acquisition, it may not be surprising that it is robust across sexes at the onset of language experience.

## Methods

### Data

#### Participants

We aggregated seven studies conducted in three laboratories on young, typically developing infants’ processing of repetition- and non-repetition- (i.e. diversity-)based regularities tested with NIRS. These studies were identified using a Google Scholar search with the terms “repetition-based regularity”, “NIRS”, “infant”. Papers including more than one study were considered separate studies. Of the 43 hits, those that did not meet the selection criteria (e.g. studies with atypical infants, behavioral studies etc.) were discarded, leaving 12 published studies. 9 further unpublished studies from the last author’s laboratory were added. Of these, studies for which information on participants’ sex was not available were discarded. The final sample comprised 7 studies with a total sample size of 150 infants (72 M, 78 F; 91 newborns, 59 six-month-olds). Information about included studies and their characteristics is given in Table 1.

#### Materials

All included studies share the use of two artificial grammars, repetition-based and non-repetition-based bisyllabic or trisyllabic auditory sequences. The specific structures employed in each study are described in Table 1. Details about the materials and stimuli can be found in the respective publications.

#### Procedure

Infants were tested with a CW-NIRS device (brand, wavelengths and sampling frequencies listed in Table 1 while sound stimuli were administered through two loud-speakers. Eight-ten sources and eight detectors were placed on infants’ heads bilaterally with a 3 cm source-detector distance, forming 10–12 channels per hemisphere (Fig. 1b). Details about the specific procedures of each study can be found in the corresponding publications.

### Data analysis

#### fNIRS pre-processing

fNIRS data was processed largely in the same way as in the original studies. Briefly, light intensities were first converted to optical densities and then to HbO and HbR concentration changes, using the modified Beer-Lambert Law with the following absorption coefficients (µ_a,_ mm^−1^·mM^−1^): µ_a_(HbO, 695 nm) = 0.0955, µ_a_(HbO, 760 nm) = 0.1496, µ_a_(HbO, 830 nm) = 0.2320, µ_a_(HbO, 850 nm) = 0.2526; µ_a_(HbR, 695 nm) = 0.4513, µ_a_(HbR, 760 nm) = 0.3865, µ_a_(HbR, 830 nm) = 0.1792 and µ_a_(HbR, 850 nm) = 0.1798.

The product of the optical pathlength and the differential pathlength factor was set to 1, so that the resulting concentrations were expressed in mM x mm. Then, data was bandpass filtered between 0.01 and 0.7 Hz, using a *fft* digital filter. Single blocks were rejected if the light intensity reached the saturation value, if they contained motion artifacts, or both. Artifacts were defined as concentration changes larger than 0.1 mM × mm over 0.2 s^24,44^. This procedure was performed on each channel independently. Channels with fewer than 20% valid blocks were discarded entirely from the analysis (M: 3.36, STD: 3.9). For the non-rejected channels, per each experiment, an average of 40.1% of blocks were discarded for poor data quality (STD: 17.9%). Rejection was carried out in batch for all infants and all studies, before the statistical analyses were performed. For the non-rejected blocks, a baseline was linearly fit between the means of the 5 s preceding the onset of the block and the 5 s preceding the onset of the next one. This pre-processing routine has been shown to yield an accurate recovery of the infant hemodynamic response^54^.

After pre-processing, channel-wise block averages were computed for each condition. Finally, grand averages were obtained by computing the average and standard errors of repetition blocks and non-repetition blocks across all studies.

#### Calculation of effect sizes

Two complementary analytic frameworks were employed: meta-analysis and mixed-effects modelling. For the meta-analysis, the effect size was calculated for each study, then its weighted average and variability across studies was estimated. This allowed to define a standardized, overall effect size for the neural manifestation of the rule-learning mechanism. Within this framework, sex differences could be analyzed at the group level. The linear mixed effects model conducted over individual-level effect sizes investigated how sex moderates the response to repetition- and non repetition-sequences and interacts with other factors, like age and brain regions. This approach has been described in detail in^34^

In particular, for each study and for each participant, activation was computed by averaging the amplitude of the response across trials of the same conditions in a time window starting at the onset of the stimulus and lasting up to 15 s after the end of the stimulation block. Activation was computed for (i) the repetition condition with respect to the zero baseline (R vs. 0), (ii) the non-repetition condition with respect to the zero baseline (N vs. 0), as well as (iii) comparing the repetition and non-repetition conditions (R vs. N), as the difference between the mean activations for repetition and the non-repetition conditions (Fig. 2).

For each of the three comparisons (R vs. 0, N vs. 0 and R vs. N), the infant-level effect size *d*_*infant*_ was computed by dividing the mean response by the standard deviation of responses across trials^55^. The study-level effect size *d*_*study*_ was computed by dividing the average of the individual responses by their standard deviation across participants. The effect size sampling variances, referring to the extent to which effect sizes are expected to vary from study to study^56^, were computed as *V*_*d*_ = 2/n + *d*_*study*_^2^/4n, with *n* being the number of participants^57^; i.e. effect sizes were weighted by the number of participants in a study^31^.

Individual and meta-analytic effect sizes were calculated for each channel and hemoglobin component.

Subsequent analyses were carried out separately for the three contrasts of interest outlined above (R vs 0, N vs 0 and R vs N), as they address different theoretical questions. While the R vs N contrast more directly represents infants’ ability to discriminate repetition-based from non-repetition-based sequences, comparisons of each condition against the baseline allows to estimate the ability of the brain to represent and process the two types of regularities independently of one another. This is relevant, because the ability to represent repetition-based and non-repetition-based, i.e. diversity-based, sequences do not emerge at the same developmental time, and constitute different underlying abilities. Most importantly, brain responses to non-repetitions, measured through the N vs 0 contrast, are larger in 6-month-olds than in newborns, and this has been recently proposed to support the beginning of word learning, an ability that indeed starts emerging at around 6 months of age^27^.

#### Selection of ROIs

Regions of interest over which activations are calculated are typically defined either anatomically or functionally. We have implemented both approaches since no a priori hypotheses were available as to whether sex differences may be observed in different anatomical regions of the brain or in hemodynamic response patterns.

##### Anatomically defined regions of interest

Four regions of interest were chosen covering the classical language network: the bilateral temporal areas (channels 3, 6 in the LH and 17, 19 in the RH, Fig. 1b), known to be responsible for auditory processing, and the bilateral frontal areas (channels 2, 5 in the LH and 13, 15 in the RH), responsible for the computation of structure and higher-order linguistic/sequential representations, following Gervain et al.^24,44^. For each participant, effect sizes were averaged across channels within each ROI. This approach allowed us to test whether sex differences exist in the responses to repetition-based structures in the classical speech and language areas.

##### Functionally defined regions of interest

Since different studies may show effects in different brain areas, for instance due to differences in the stimuli, the headgear used or the ages tested, we also carried out a data-driven functional localization analysis in order to identify clusters of channels that significantly activate for repetition and non-repetition sequences in each study.

These functional ROIs were identified using cluster-based permutation tests involving t-tests^45^ over oxygenated hemoglobin (HbO) concentrations, i.e. the chromophore that shows stronger effects in infants^29^. Separate permutation tests were conducted for all three contrasts (R vs. 0, N vs. 0, R vs. N). Statistical significance was assessed against the null distribution of *t* values obtained by randomly relabelling data (1000 iterations), as is now standard in infant NIRS studies^30,58,59^. For each study, the strongest cluster (i.e. having the largest *t* value) was selected in each hemisphere. The functionally defined ROIS were used in the linear mixed effects model.

#### Statistical analysis

##### Meta-analysis

Study-level effect sizes were analysed by fitting a meta-analytic random-effects model with the *metafor* R package^60,61^. Models were fit using restricted maximum likelihood (REML). A model was fit over the entire dataset, while models were also applied to the four anatomically defined ROIs separately. After establishing the overall effect sizes for all babies confounded, similar analyses were run with Sex as a moderator to test for sex differences.

#### Linear mixed effects model

##### Anatomically defined regions of interest

Linear mixed effects models were carried out over individual-level effect sizes separately for newborns and six months olds as there are known developmental changes in the processing of non-repetition patterns^27^. The random-effects structure consisted of random intercepts for participant, study and lab. Candidate fixed effects included Sex (female/male), ROI (temporal/frontal), Hemisphere (LH/RH) as well as their interactions. They were incrementally included in the fixed-effects structure and the resulting models were compared. For each contrast and age group, the best fitting model was chosen based on the AIC (Akaike information criterion) value.

#### Functionally defined regions of interest

Models were again fit separately for the two age groups. The planned random-effects structure consisted of random intercepts for participant, study and lab. Candidate fixed effects included Sex (female/male) and Hemisphere (LH/RH) as well as their interaction. Model selection was performed by selecting the best fitting model based on the AIC (Akaike information criterion) value.

For both analyses, models were fit using the *lmer* function from the *lme4* R package^60,62^, with denominator degrees of freedom being estimated with the Kenward-Roger method.

### Supplementary Information

Supplementary Information.

## Data Availability

The datasets used and/or analysed in the current study are available from the corresponding author on reasonable request.

## References

[1] Frith U (2001). Mind blindness and the brain in autism. Neuron.

[2] Miles TR, Haslum MN, Wheeler TJ (1998). Gender ratio in dyslexia. Ann. Dyslexia.

[3] Halpern DF (2013). Sex Differences in Cognitive Abilities.

[4] Wallentin M (2009). Putative sex differences in verbal abilities and language cortex: A critical review. Brain Lang..

[5] Fenson L (1994). Variability in early communicative development source. Monogr. Soc. Res. Child Dev..

[6] Eriksson M (2012). Differences between girls and boys in emerging language skills: Evidence from 10 language communities. Br. J. Dev. Psychol..

[7] Bouchard C, Trudeau N, Sutton A, Boudreault MC, Deneault J (2009). Gender differences in language development in French Canadian children between 8 and 30 months of age. Appl. Psycholinguist..

[8] Galsworthy MJ, Dionne G, Dale PS, Plomin R (2000). Sex differences in early verbal and non-verbal cognitive development. Dev. Sci..

[9] De Bellis MD (2001). Sex differences in brain maturation during childhood and adolescence. Cereb. Cortex.

[10] Lenroot RK (2007). Sexual dimorphism of brain developmental trajectories during childhood and adolescence. NeuroImage.

[11] Ingalhalikar M (2014). Sex differences in the structural connectome of the human brain. Proc. Natl. Acad. Sci. U. S. A..

[12] Etchell A (2016). A systematic literature review of sex differences in childhood language and brain development Andrew. Physiol. Behav..

[13] Geschwind N, Galaburda AM (1985). Cerebral lateralization. Biological mechanisms, associations, and pathology: I. A hypothesis and a program for research. Arch. Neurol..

[14] Wheelock MD (2019). Sex differences in functional connectivity during fetal brain development. Dev. Cogn. Neurosci..

[15] Leaper C, Bornstein MH (2002). Parenting girls and boys. Handbook of parenting: Children and parenting.

[16] Bornstein MH, Hahn C, Haynes OM (2004). Specific and general language performance across early childhood: Stability and gender considerations. First Lang..

[17] Barbu S (2015). Sex differences in language across early childhood: Family socioeconomic status does not impact boys and girls equally. Front. Psychol..

[18] Farrant BM, Mattes E, Keelan JA, Hickey M, Whitehouse AJO (2013). Fetal testosterone, socio-emotional engagement and language development. Infant Child Dev..

[19] Rich-Edwards JW, Kaiser UB, Chen GL, Manson JAE, Goldstein JM (2018). Sex and gender differences research design for basic, clinical, and population studies: Essentials for investigators. Endocr. Rev..

[20] Maccoby EE, Jacklin CN (1978). The Psychology of Sex Differences.

[21] Zambrana IM, Ystrom E, Pons F (2012). Impact of gender, maternal education, and birth order on the development of language comprehension: A longitudinal study from 18 to 36 months of age. J. Dev. Behav. Pediatr..

[22] Le Normand M, Parisse C, Cohen H (2008). Lexical diversity and productivity in French preschoolers: Developmental, gender and sociocultural factors. Clin. Linguist. Phon..

[23] Heidari S, Babor TF, De Castro P, Tort S, Curno M (2016). Sex and Gender Equity in Research: Rationale for the SAGER guidelines and recommended use. Res. Integr. Peer Rev..

[24] Gervain J, Berent I, Werker JF (2012). Binding at birth: The newborn brain detects identity relations and sequential position in speech. J. Cognit. Neurosci..

[25] Gervain J, de la Cruz-Pavía I, Gerken LA (2020). Behavioral and imaging studies of infant artificial grammar learning. Top. Cognit. Sci..

[26] Rabagliati H, Ferguson B, Lew-Williams C (2019). The profile of abstract rule learning in infancy: Meta-analytic and experimental evidence. Dev. Sci..

[27] de la Cruz-Pavía I, Gervain J (2021). Infants’ perception of repetition-based regularities in speech: A look from the perspective of the same/different distinction. Curr. Opin. Behav. Sci..

[28] Boas DA, Elwell CE, Ferrari M, Taga G (2014). Twenty years of functional near-infrared spectroscopy: Introduction for the special issue. NeuroImage.

[29] Gervain J (2011). Near-infrared spectroscopy: A report from the McDonnell infant methodology consortium. Dev. Cognit. Neurosci..

[30] Abboub N, Nazzi T, Gervain J (2016). Prosodic grouping at birth. Brain Lang..

[31] Bergmann C (2018). Promoting replicability in developmental research through meta-analyses: Insights from language acquisition research. Child Dev..

[32] Lewis, M. *et al.* A quantitative synthesis of early language acquisition using meta-analysis. 1–24 (2018). 10.17605/OSF.IO/HTSJM

[33] Baek, S. *et al.* Attrition rate in infant fNIRS research: A meta-analysis. *bioRxiv* (2021). 10.1111/infa.12521 36748788

[34] Gemignani J (2023). Reproducibility of infant fNIRS studies: A meta-analytic approach. Neurophotonics.

[35] Gervain J, Minagawa Y, Emberson L, Lloyd-Fox S (2023). Using functional near-infrared spectroscopy to study the early developing brain: Future directions and new challenges. Neurophotonics.

[36] Valentine JC, Pigott TD, Rothstein HR (2010). How many studies do you need? A primer on statistical power for meta-analysis. J. Educ. Behav. Stat..

[37] Braver SL, Thoemmes FJ, Rosenthal R (2014). Continuously cumulating meta-analysis and replicability. Perspect. Psychol. Sci..

[38] Goh JX, Hall JA, Rosenthal R (2016). Mini meta-analysis of your own studies: Some arguments on why and a primer on how: Mini meta-analysis. Soc. Pers. Psychol. Compass.

[39] Maner JK (2014). Let’s put our money where our mouth is: If authors are to change their ways, reviewers (and editors) must change with them. Perspect. Psychol. Sci..

[40] Cumming G (2014). The new statistics: Why and how. Psychol. Sci..

[41] Bouchon, C. Functional asymmetry between consonants and vowels from birth to 6 months of age: Cerebral imaging and behavioral data. Psychology. Université Paris Descartes (Paris 5), English (2014).

[42] Bouchon C, Nazzi T, Gervain J (2015). Hemispheric asymmetries in repetition enhancement and suppression effects in the newborn brain. PLoS ONE.

[43] Berent I, de la Cruz-Pavía I, Brentari D, Gervain J (2021). Infants differentially extract rules from language. Sci. Rep..

[44] Gervain J, Macagno F, Cogoi S, Pena M, Mehler J (2008). The neonate brain detects speech structure. Proc. Natl. Acad. Sci..

[45] Maris E, Oostenveld R (2007). Nonparametric statistical testing of EEG- and MEG-data. J. Neurosci. Methods.

[46] Lin PY (2013). Regional and hemispheric asymmetries of cerebral hemodynamic and oxygen metabolism in newborns. Cereb. Cortex.

[47] Lloyd-Fox S, Blasi A, Elwell CE (2010). Illuminating the developing brain: The past, present and future of functional near infrared spectroscopy. Neurosci. Biobehav. Rev..

[48] Di Lorenzo R (2019). Recommendations for motion correction of infant fNIRS data applicable to multiple data sets and acquisition systems. NeuroImage.

[49] *Studies on Reduplication*. [De Gruyter Mouton, 2005]. 10.1515/9783110911466

[50] Tanaka C, Matsui M, Uematsu A, Noguchi K, Miyawaki T (2012). Developmental trajectories of the fronto-temporal lobes from infancy to early adulthood in healthy individuals. Dev. Neurosci..

[51] Dehaene-Lambertz G, Spelke ES (2015). The infancy of the human brain. Neuron.

[52] Leroy F (2011). Early maturation of the linguistic dorsal pathway in human infants. J. Neurosci..

[53] Dehaene-Lambertz G, Dehaene S, Hertz-Pannier L (2002). Functional neuroimaging of speech perception in infants. Science.

[54] Gemignani J, Gervain J (2021). Comparing different pre-processing routines for infant fNIRS data. Dev. Cognit. Neurosci..

[55] Frost RLA (2020). Non-adjacent dependency learning in infancy, and its link to language development. Cognit. Psychol..

[56] Morris SB, DeShon RP (2002). Combining effect size estimates in meta-analysis with repeated measures and independent-groups designs. Psychol. Methods.

[57] Borenstein M, Hedges LV, Higgins JPT, Rothstein HR (2009). Introduction to Meta-Analysis. Introd. Meta-Anal.

[58] Cabrera L, Gervain J (2020). Speech perception at birth: The brain encodes fast and slow temporal information. Sci. Adv..

[59] Ferry AL (2016). On the edge of language acquisition: Inherent constraints on encoding multisyllabic sequences in the neonate brain. Dev. Sci..

[60] R Core Team. R: A Language and Environment for Statistical Computing. (2022). <https://www.r-project.org/>

[61] Viechtbauer W (2010). Conducting meta-analyses in R with the metafor. J. Stat. Softw..

[62] Bates D, Mächler M, Bolker BM, Walker SC (2015). Fitting linear mixed-effects models using lme4. J. Stat. Softw..

