# Supplementary material for: Brain responses to repetition-based rule-learning do not exhibit sex differences: an aggregated analysis of infant fNIRS studies
Source: Sci Rep. 2024 Jan 31;14:2611. doi: 10.1038/s41598-024-53092-2 (PMC10831066; doi:10.1038/s41598-024-53092-2)
Supplement: Supplementary file 1 — Supplementary Information. [file 41598_2024_53092_MOESM1_ESM.pdf]

Brain responses to repetition-based rule-learning do not exhibit sex differences:

an aggregated analysis of infant fNIRS studies

Supplementary Material

Jessica Gemignani[1, 2, 3], Judit Gervain[1, 2, 3]

[1] Department of Developmental and Social Psychology, University of Padua, Padua, Italy

[2] Padova Neuroscience Center, University of Padua, Padua, Italy

[3] Integrative Neuroscience and Cognition Center, CNRS & Université Paris Cité, Paris, France

RESULTS

1. **Meta-analysis**

1.1.Estimates of the effects (intercept-only models)

Below are listed the results, for each comparison R>0, N>0 and R>N, of the intercept-only models, i.e. across regions of interest, age groups and sexes. Results of these models, for HbO, are reported in Section 3.1: "Estimates of the effects".

	**R vs 0, HbO**	**R vs 0, HbR**	**N vs 0, HbO**	**N vs 0, HbR**	**R vs N, HbO**	**R vs N, HbR**
**tau^2**	0.000	0.000	0.000	0.000	0.000	0.000
**tau^2 - SE**	0.026	0.025	0.025	0.025	0.025	0.025
**Tau**	0.000	0.000	0.000	0.000	0.000	0.000
**I^2**	0.000	0.000	0.000	0.000	0.000	0.000
**H^2**	1.000	1.000	1.000	1.000	1.000	1.000
**Q**	22.234	14.566	13.957	10.177	18.221	13.775
**Qp**	0.725	0.975	0.982	0.999	0.897	0.983
**estimate**	0.256	-0.169	0.131	-0.189	0.123	0.005
**SE**	0.058	0.058	0.058	0.058	0.058	0.058
**z**	4.410	-2.921	2.264	-3.262	2.122	0.085
p	**0.000**	**0.003**	**0.024**	**0.001**	**0.034**	0.932
**CI – low**	0.142	-0.283	0.018	-0.302	0.009	-0.108
**CI - upp**	0.370	-0.056	0.000	-0.075	0.236	0.118

Table S1: Results of the intercept-only random effects meta-analytic models for all contrasts, for both hemoglobin components; highlighted in bold are significant *p* values at $\alpha = 0.05$.

1.2 Subgroup models

The following tables present a summary of the meta-analytic models carried out in this work. Each table is divided in the three comparisons: R>0, N>0 and R>N, that were analysed independently. Within each comparison, cells marked with "**M+F**" on the row and as **"Both"** in column report the results of the overall RE Model reported in text at the bottom of the forest plots in Figures 3, 4 and 5 (HbO) and Figures S1, S2 and S3 (HbR).

Additionally, for each ROI three subgroup analyses were carried out for the two age groups, newborns and six months:

- Subgroup analysis newborns vs six-month-olds for males only (columns: **0m, 6m,** row: **"M"**)
- Subgroup analysis newborns vs six-month-olds for females only (columns: **0m, 6m,** row: **"F"**)
- Subgroup analysis newborns vs six-month-olds across sexes (columns: **0m, 6m,** row: **"M+F"**).

Results of all the subgroup analyses are also reported in the forest plots in Figures 3, 4 and 5. Subgroup analyses were carried out to estimate the effect

size to each condition within the specific categories. Statistical analysis of sex differences was carried out with moderated analyses, described in the next section.

In the tables that follow, we highlighted cells according to the following color coding:

Statistical significance of **effect sizes *("p")***

- Cells highlighted in YELLOW = significant effect at $p < 0.05$
- Cells highlighted in ORANGE= marginal effect ($p < 0.1$)

Statistical significance of **heterogeneity (**"***Qp***")

- Cells highlighted in GREEN = significant heterogeneity ($p < 0.05$)
- Cells highlighted in FAINT GREEN = marginal heterogeneity ($p < 0.1$)
- Cells highlighted in GREY = marginal or significant residual heterogeneity after including moderator ($p < 0.1$)

1.3 Sex differences: moderated models

Within each ROI, three moderated analyses were carried out with Sex as moderator:

- Moderated analysis for newborns only (column: **0m**)
- Moderated analysis for six-month-olds only (column: **6m**)
- Moderated analysis across age groups (column: **Both**)

In the tables that follow, we highlighted cells according to the following color coding:

Statistical significance of moderating variable ("***QMp*"**)

- Cells highlighted in CYAN = significant effect of Sex ($p < 0.05$); *note: no significant effects were found*

Statistical significance of residual heretogeneity ("***QEp***")

- Cells highlighted in GREY = marginal heterogeneity ($p < 0.1$)

Frontal LH, HbO

	N>0			R>0			R>N		
	**0m**	**6m**	**Both**	**0m**	**6m**	**Both**	**0m**	**6m**	**Both**
**M+F**									
tau^2	0.000	0.000	0.000	0.019	0.037	0.010	0.000	0.000	0.000
tau	0.000	0.000	0.000	0.138	0.193	0.100	0.000	0.000	0.000
I^2	0.000	0.000	0.000	17.790	26.185	9.542	0.000	0.000	0.000
H^2	1.000	1.000	1.000	1.216	1.355	1.105	1.000	1.000	1.000
Q	0.696	0.590	4.538	3.637	2.599	6.366	1.116	0.811	3.571
Qp	0.874	0.744	0.604	0.303	0.273	0.383	0.773	0.667	0.735
**Estimate**	**-0.123**	**0.305**	**0.043**	**0.100**	**0.178**	**0.132**	**0.264**	**-0.040**	**0.145**
SE	0.148	0.186	0.116	0.164	0.217	0.122	0.148	0.185	0.116
**Z**	-0.832	1.643	0.372	0.612	0.817	1.082	1.777	-0.217	1.250
**P**	0.406	0.100	0.710	0.541	0.414	0.279	0.076	0.828	0.211
**ci.lb**	-0.413	-0.059	-0.184	-0.221	-0.248	-0.107	-0.027	-0.402	-0.082
**ci.ub**	0.167	0.669	0.270	0.421	0.603	0.371	0.554	0.322	0.371
**M**									
tau^2	0.000	0.050		0.000	0.000		0.000	0.000	
tau	0.000	0.223		0.000	0.000		0.000	0.000	
I^2	0.000	18.822		0.000	0.000		0.000	0.000	
H^2	1.000	1.232		1.000	1.000		1.000	1.000	
Q	0.849	2.333		1.278	1.012		0.532	0.469	
Qp	0.838	0.311		0.734	0.603		0.912	0.791	
**Estimate**	**-0.150**	**0.161**		**0.069**	**0.327**		**0.167**	**0.332**	
SE	0.217	0.296		0.217	0.266		0.217	0.266	
Z	-0.690	0.544		0.320	1.228		0.771	1.250	
**P**	0.490	0.587		0.749	0.219		0.441	0.211	
ci.lb	-0.575	-0.420		-0.355	-0.195		-0.258	-0.189	
ci.ub	0.276	0.742		0.494	0.848		0.592	0.854	
**F**									
tau^2	0.000	0.000		0.070	0.000		0.000	0.000	
tau	0.000	0.000		0.264	0.000		0.000	0.000	
I^2	0.000	0.000		28.850	0.000		0.000	0.000	
H^2	1.000	1.000		1.405	1.000		1.000	1.000	
Q	0.273	0.019		4.112	1.543		1.843	0.343	
Qp	0.965	0.990		0.250	0.462		0.606	0.842	
Estimate	-0.039	0.481		0.041	0.121		0.126	-0.198	
SE	0.205	0.263		0.246	0.261		0.206	0.260	
Z	-0.189	1.832		0.168	0.463		0.611	-0.762	
**P**	0.850	0.067		0.866	0.643		0.541	0.446	
ci.lb	-0.441	-0.034		-0.441	-0.390		-0.278	-0.708	
ci.ub	0.363	0.996		0.524	0.632		0.529	0.312	

Moderated analysis: SEX

	N>0			R>0			R>N		
	**0m**	**6m**	**Both**	**0m**	**6m**	**Both**	**0m**	**6m**	**Both**
**QE**	1.122	2.352	6.739	5.389	2.555	8.584	2.375	0.812	4.373
**QEdf**	6.000	4.000	12.000	6.000	4.000	12.000	6.000	4.000	12.000
**QEp**	0.981	0.671	0.874	0.495	0.635	0.738	0.882	0.937	0.976
**QM**	0.138	0.721	0.615	0.019	0.305	0.214	0.019	2.034	0.993
**QMdf**	1.000	1.000	1.000	1.000	1.000	1.000	1.000	1.000	1.000
**QMp**	0.710	0.396	0.433	0.891	0.581	0.644	0.890	0.154	0.319

Frontal LH, HbR

	N>0			R>0			R>N		
	**0m**	**6m**	**Both**	**0m**	**6m**	**Both**	**0m**	**6m**	**Both**
**M+F**									
tau^2	0.000	0.000	0.000	0.000	0.000	0.000	0.000	0.000	0.000
tau	0.000	0.000	0.000	0.000	0.000	0.000	0.000	0.000	0.000
I^2	0.000	0.000	0.000	0.000	0.000	0.000	0.000	0.000	0.000
H^2	1.000	1.000	1.000	1.000	1.000	1.000	1.000	1.000	1.000
Q	2.733	0.244	3.228	0.345	0.766	3.004	1.989	0.142	4.413
Qp	0.435	0.885	0.780	0.951	0.682	0.808	0.575	0.931	0.621
**Estimate**	**-0.100**	**-0.219**	**-0.146**	**-0.256**	**0.071**	**-0.128**	**-0.154**	**0.204**	**-0.013**
SE	0.148	0.185	0.116	0.148	0.185	0.116	0.149	0.185	0.116
**Z**	-0.673	-1.182	-1.265	-1.723	0.382	-1.105	-1.037	1.104	-0.116
**P**	0.501	0.237	0.206	0.085	0.703	0.269	0.300	0.270	0.908
**ci.lb**	-0.391	-0.581	-0.373	-0.546	-0.292	-0.355	-0.446	-0.158	-0.240
**ci.ub**	0.191	0.144	0.080	0.035	0.433	0.099	0.137	0.566	0.214
**M**									
tau^2	0.079	0.000		0.000	0.000		0.000	0.000	
tau	0.281	0.000		0.000	0.000		0.000	0.000	
I^2	28.847	0.000		0.000	0.000		0.000	0.000	
H^2	1.405	1.000		1.000	1.000		1.000	1.000	
Q	4.016	0.027		0.536	0.103		1.806	0.099	
Qp	0.260	0.987		0.911	0.950		0.614	0.952	
**Estimate**	**-0.120**	**-0.407**		**-0.293**	**-0.022**		**-0.137**	**0.279**	
SE	0.261	0.266		0.218	0.263		0.218	0.264	
Z	-0.461	-1.529		-1.346	-0.082		-0.627	1.054	
**P**	0.644	0.126		0.178	0.934		0.531	0.292	
ci.lb	-0.632	-0.928		-0.719	-0.538		-0.564	-0.239	
ci.ub	0.391	0.115		0.134	0.494		0.291	0.796	
**F**									
tau^2	0.000	0.000		0.000	0.000		0.000	0.000	
tau	0.000	0.000		0.002	0.000		0.000	0.000	
I^2	0.000	0.000		0.003	0.000		0.000	0.000	
H^2	1.000	1.000		1.000	1.000		1.000	1.000	
Q	1.248	0.169		3.182	0.395		0.729	0.081	
Qp	0.742	0.919		0.364	0.821		0.866	0.960	
**Estimate**	**-0.151**	**-0.232**		**-0.237**	**0.022**		**-0.105**	**0.102**	
SE	0.206	0.260		0.207	0.259		0.206	0.259	
Z	-0.733	-0.892		-1.145	0.085		-0.509	0.392	
**P**	0.464	0.372		0.252	0.933		0.611	0.695	
ci.lb	-0.554	-0.742		-0.644	-0.486		-0.509	-0.407	
ci.ub	0.252	0.278		0.169	0.530		0.299	0.610	

Moderated analysis: SEX

	N>0			R>0			R>N		
	**0m**	**6m**	**Both**	**0m**	**6m**	**Both**	**0m**	**6m**	**Both**
**QE**	5.264	0.195	6.268	3.718	0.498	5.457	2.535	0.179	4.573
**QEdf**	6.000	4.000	12.000	6.000	4.000	12.000	6.000	4.000	12.000
**QEp**	0.510	0.996	0.902	0.715	0.974	0.941	0.864	0.996	0.971
**QM**	0.020	0.220	0.041	0.034	0.014	0.040	0.011	0.228	0.059
**QMdf**	1.000	1.000	1.000	1.000	1.000	1.000	1.000	1.000	1.000
**QMp**	0.888	0.639	0.840	0.854	0.906	0.841	0.916	0.633	0.808

Temporal LH, HbO									
	N>0			R>0			R>N		
	**0m**	**6m**	**Both**	**0m**	**6m**	**Both**	**0m**	**6m**	**Both**
**M+F**									
tau^2	0.000	0.000	0.000	0.031	0.000	0.000	0.185	0.000	0.064
tau	0.000	0.000	0.000	0.175	0.000	0.000	0.430	0.000	0.252
I^2	0.000	0.000	0.000	25.499	0.000	0.000	67.238	0.000	40.121
H^2	1.000	1.000	1.000	1.342	1.000	1.000	3.052	1.000	1.670
Q	1.098	0.736	2.172	4.048	0.268	4.464	9.131	0.056	9.958
Qp	0.778	0.692	0.903	0.256	0.875	0.614	0.028	0.973	0.126
**Estimate**	**0.097**	**0.235**	**0.151**	**0.359**	**0.262**	**0.318**	**0.204**	**-0.024**	**0.106**
SE	0.148	0.185	0.116	0.174	0.185	0.116	0.262	0.185	0.151
**Z**	0.659	1.268	1.306	2.069	1.414	2.728	0.780	-0.133	0.703
**P**	0.510	0.205	0.192	0.039	0.157	0.006	0.435	0.894	0.482
**ci.lb**	-0.192	-0.128	-0.076	0.019	-0.101	0.089	-0.309	-0.386	-0.190
**ci.ub**	0.387	0.599	0.378	0.699	0.625	0.546	0.718	0.337	0.402
**M**									
tau^2	0.010	0.000		0.000	0.000		0.130	0.000	
tau	0.098	0.000		0.000	0.000		0.360	0.000	
I^2	4.608	0.000		0.000	0.000		39.694	0.000	
H^2	1.048	1.000		1.000	1.000		1.658	1.000	
Q	3.734	1.125		1.373	0.702		5.073	1.035	
Qp	0.292	0.570		0.712	0.704		0.167	0.596	
**Estimate**	**0.347**	**-0.008**		**0.415**	**0.075**		**-0.009**	**0.056**	
SE	0.226	0.264		0.220	0.264		0.286	0.265	
Z	1.534	-0.031		1.883	0.285		-0.030	0.211	
**P**	0.125	0.976		0.060	0.775		0.976	0.833	
ci.lb	-0.096	-0.526		-0.017	-0.442		-0.570	-0.463	
ci.ub	0.790	0.510		0.847	0.593		0.552	0.574	
**F**									
tau^2	0.000	0.000		0.009	0.000		0.366	0.000	
tau	0.000	0.000		0.095	0.000		0.605	0.000	
I^2	0.000	0.000		4.945	0.000		66.968	0.000	
H^2	1.000	1.000		1.052	1.000		3.027	1.000	
Q	2.372	1.016		3.141	0.746		8.926	1.563	
Qp	0.499	0.602		0.370	0.689		0.030	0.458	
**Estimate**	**0.012**	**0.487**		**0.276**	**0.214**		**0.259**	**0.002**	
SE	0.206	0.264		0.213	0.261		0.370	0.263	
Z	0.057	1.843		1.299	0.818		0.699	0.008	
**P**	0.955	0.065		0.194	0.413		0.485	0.994	
ci.lb	-0.392	-0.031		-0.141	-0.298		-0.467	-0.514	
ci.ub	0.415	1.006		0.694	0.726		0.985	0.518	
**Moderated analysis: SEX**									
	N>0			R>0			R>N		
	**0m**	**6m**	**Both**	**0m**	**6m**	**Both**	**0m**	**6m**	**Both**
**QE**	6.106	2.141	11.304	4.513	1.448	6.969	13.999	2.598	17.026
**QEdf**	6.000	4.000	12.000	6.000	4.000	12.000	6.000	4.000	12.000
**QEp**	0.411	0.710	0.503	0.608	0.836	0.860	0.030	0.627	0.149
**QM**	1.210	1.755	0.001	0.215	0.139	0.011	0.340	0.021	0.206
**QMdf**	1.000	1.000	1.000	1.000	1.000	1.000	1.000	1.000	1.000
**QMp**	0.271	0.185	0.972	0.643	0.709	0.917	0.560	0.885	0.650

Temporal LH, HbR

	N>0			R>0			R>N		
	**0m**	**6m**	**Both**	**0m**	**6m**	**Both**	**0m**	**6m**	**Both**
**M+F**									
tau^2	0.000	0.000	0.000	0.000	0.000	0.000	0.000	0.000	0.000
tau	0.000	0.000	0.000	0.000	0.000	0.000	0.000	0.000	0.000
I^2	0.000	0.000	0.000	0.000	0.000	0.000	0.000	0.000	0.000
H^2	1.000	1.000	1.000	1.000	1.000	1.000	1.000	1.000	1.000
Q	0.114	0.179	0.745	2.876	1.514	4.707	2.059	0.540	2.648
Qp	0.990	0.914	0.993	0.411	0.469	0.582	0.560	0.763	0.852
**Estimate**	**-0.330**	**-0.171**	**-0.267**	**-0.263**	**-0.129**	**-0.211**	**0.046**	**0.098**	**0.066**
SE	0.149	0.185	0.116	0.149	0.186	0.116	0.148	0.185	0.116
**Z**	-2.222	-0.924	-2.310	-1.769	-0.696	-1.815	0.310	0.530	0.573
**P**	0.026	0.356	0.021	0.077	0.487	0.069	0.756	0.596	0.566
**ci.lb**	-0.621	-0.533	-0.494	-0.555	-0.493	-0.438	-0.244	-0.265	-0.160
**ci.ub**	-0.039	0.191	-0.041	0.028	0.235	0.017	0.336	0.460	0.293
**M**									
tau^2	0.000	0.000		0.000	0.000		0.000	0.000	
tau	0.000	0.000		0.000	0.000		0.000	0.000	
I^2	0.000	0.000		0.000	0.000		0.000	0.000	
H^2	1.000	1.000		1.000	1.000		1.000	1.000	
Q	1.430	0.819		1.762	0.779		2.184	0.478	
Qp	0.699	0.664		0.623	0.677		0.535	0.787	
**Estimate**	**-0.260**	**-0.302**		**-0.219**	**-0.310**		**0.039**	**0.123**	
SE	0.219	0.265		0.218	0.265		0.219	0.264	
Z	-1.188	-1.139		-1.004	-1.166		0.179	0.465	
**P**	0.235	0.255		0.315	0.243		0.858	0.642	
ci.lb	-0.688	-0.822		-0.645	-0.830		-0.390	-0.395	
ci.ub	0.169	0.218		0.208	0.211		0.469	0.641	
**F**									
tau^2	0.000	0.000		0.000	0.000		0.000	0.020	
tau	0.001	0.000		0.000	0.000		0.000	0.141	
I^2	0.000	0.000		0.000	0.000		0.000	8.524	
H^2	1.000	1.000		1.000	1.000		1.000	1.093	
Q	2.855	1.541		1.185	0.430		1.278	2.576	
Qp	0.415	0.463		0.757	0.806		0.734	0.276	
**Estimate**	**-0.379**	**-0.119**		**-0.295**	**-0.054**		**0.035**	**0.024**	
SE	0.208	0.262		0.206	0.261		0.205	0.277	
Z	-1.826	-0.454		-1.431	-0.208		0.172	0.086	
**P**	0.068	0.650		0.153	0.835		0.863	0.931	
ci.lb	-0.787	-0.632		-0.699	-0.566		-0.366	-0.518	
ci.ub	0.028	0.395		0.109	0.457		0.437	0.566	

Moderated analysis: SEX

	N>0			R>0			R>N		
	**0m**	**6m**	**Both**	**0m**	**6m**	**Both**	**0m**	**6m**	**Both**
**QE**	4.285	2.360	7.268	2.946	1.209	4.749	3.462	3.054	6.579
**QEdf**	6.000	4.000	12.000	6.000	4.000	12.000	6.000	4.000	12.000
**QEp**	0.638	0.670	0.839	0.816	0.877	0.966	0.749	0.549	0.884
**QM**	0.157	0.242	0.000	0.065	0.470	0.051	0.000	0.082	0.038
**QMdf**	1.000	1.000	1.000	1.000	1.000	1.000	1.000	1.000	1.000
**QMp**	0.692	0.623	0.994	0.799	0.493	0.821	0.990	0.774	0.846

Frontal RH, HbO

	N>0			R>0			R>N		
	**0m**	**6m**	**Both**	**0m**	**6m**	**Both**	**0m**	**6m**	**Both**
**M+F**									
tau^2	0.000	0.000	0.000	0.000	0.000	0.000	0.000	0.000	0.000
tau	0.000	0.000	0.000	0.000	0.000	0.000	0.000	0.000	0.000
I^2	0.000	0.000	0.000	0.000	0.000	0.000	0.000	0.000	0.000
H^2	1.000	1.000	1.000	1.000	1.000	1.000	1.000	1.000	1.000
Q	0.440	1.676	3.025	1.311	0.945	3.187	1.664	0.276	1.942
Qp	0.932	0.433	0.806	0.726	0.623	0.785	0.645	0.871	0.925
**Estimate**	**-0.031**	**0.195**	**0.057**	**0.066**	**0.295**	**0.155**	**0.119**	**0.108**	**0.115**
SE	0.148	0.185	0.115	0.148	0.186	0.116	0.148	0.184	0.116
**Z**	-0.209	1.054	0.494	0.448	1.591	1.342	0.801	0.586	0.992
**P**	0.835	0.292	0.622	0.654	0.112	0.180	0.423	0.558	0.321
**ci.lb**	-0.320	-0.168	-0.169	-0.224	-0.068	-0.071	-0.172	-0.254	-0.112
**ci.ub**	0.259	0.558	0.283	0.356	0.659	0.382	0.409	0.470	0.341
**M**									
tau^2	0.000	0.000		0.000	0.000		0.000	0.000	
tau	0.000	0.000		0.000	0.000		0.000	0.000	
I^2	0.000	0.000		0.000	0.000		0.000	0.000	
H^2	1.000	1.000		1.000	1.000		1.000	1.000	
Q	1.225	0.774		1.410	0.241		2.337	0.380	
Qp	0.747	0.679		0.703	0.886		0.505	0.827	
**Estimate**	**-0.062**	**0.113**		**0.043**	**0.399**		**0.082**	**0.258**	
SE	0.217	0.264		0.217	0.267		0.218	0.265	
Z	-0.288	0.428		0.199	1.497		0.375	0.976	
**P**	0.773	0.669		0.843	0.134		0.708	0.329	
ci.lb	-0.487	-0.405		-0.382	-0.124		-0.345	-0.261	
ci.ub	0.362	0.631		0.468	0.922		0.508	0.777	
**F**									
tau^2	0.000	0.000		0.000	0.000		0.000	0.000	
tau	0.000	0.000		0.000	0.000		0.000	0.000	
I^2	0.000	0.000		0.000	0.000		0.000	0.000	
H^2	1.000	1.000		1.000	1.000		1.000	1.000	
Q	0.617	0.011		0.861	0.065		0.541	0.022	
Qp	0.893	0.994		0.835	0.968		0.910	0.989	
**Estimate**	**-0.092**	**0.425**		**0.031**	**0.354**		**0.192**	**-0.068**	
SE	0.205	0.262		0.205	0.262		0.205	0.259	
Z	-0.451	1.622		0.149	1.351		0.936	-0.264	
**P**	0.652	0.105		0.882	0.177		0.349	0.792	
ci.lb	-0.494	-0.089		-0.371	-0.159		-0.210	-0.577	
ci.ub	0.309	0.938		0.433	0.867		0.594	0.440	
**Moderated analysis: SEX**									
	**N>0**			**R>0**			**R>N**		
	**0m**	**6m**	**Both**	**0m**	**6m**	**Both**	**0m**	**6m**	**Both**
**QE**	1.842	0.785	5.307	2.271	0.307	4.596	2.878	0.402	4.166
**QEdf**	6.000	4.000	12.000	6.000	4.000	12.000	6.000	4.000	12.000
**QEp**	0.934	0.940	0.947	0.893	0.989	0.970	0.824	0.982	0.980
**QM**	0.010	0.701	0.170	0.002	0.015	0.018	0.136	0.778	0.069
**QMdf**	1.000	1.000	1.000	1.000	1.000	1.000	1.000	1.000	1.000
**QMp**	0.920	0.402	0.680	0.967	0.903	0.894	0.712	0.378	0.793

Frontal RH, HbR									
	N>0			R>0			R>N		
	**0m**	**6m**	**Both**	**0m**	**6m**	**Both**	**0m**	**6m**	**Both**
**M+F**									
tau^2	0.000	0.000	0.000	0.000	0.000	0.000	0.000	0.000	0.000
tau	0.000	0.000	0.000	0.000	0.018	0.000	0.000	0.000	0.000
I^2	0.000	0.000	0.000	0.000	0.326	0.000	0.000	0.000	0.000
H^2	1.000	1.000	1.000	1.000	1.003	1.000	1.000	1.000	1.000
Q	1.422	0.312	2.065	1.177	1.860	3.053	2.053	0.606	1.422
Qp	0.700	0.856	0.914	0.759	0.394	0.802	0.561	0.739	0.700
**Estimate**	**-0.073**	**-0.209**	**-0.126**	**-0.117**	**-0.087**	**-0.106**	**-0.014**	**0.062**	**-0.073**
SE	0.148	0.185	0.115	0.148	0.185	0.116	0.148	0.185	0.148
**Z**	-0.492	-1.130	-1.091	-0.794	-0.469	-0.914	-0.097	0.338	-0.492
**P**	0.622	0.258	0.275	0.427	0.639	0.361	0.923	0.735	0.622
**ci.lb**	-0.363	-0.571	-0.352	-0.407	-0.451	-0.332	-0.305	-0.300	-0.363
**ci.ub**	0.217	0.153	0.100	0.173	0.276	0.121	0.276	0.424	0.217
**M**									
tau^2	0.015	0.000		0.000	0.000		0.110	0.000	
tau	0.123	0.000		0.000	0.000		0.331	0.000	
I^2	7.324	0.000		0.000	0.000		36.182	0.000	
H^2	1.079	1.000		1.000	1.000		1.567	1.000	
Q	2.967	0.436		1.288	0.924		4.610	0.961	
Qp	0.397	0.804		0.732	0.630		0.203	0.618	
**Estimate**	**0.067**	**-0.416**		**-0.231**	**-0.284**		**-0.197**	**-0.059**	
SE	0.227	0.266		0.217	0.266		0.276	0.264	
Z	0.295	-1.561		-1.062	-1.069		-0.714	-0.225	
**P**	0.768	0.119		0.288	0.285		0.475	0.822	
ci.lb	-0.378	-0.938		-0.657	-0.804		-0.737	-0.577	
ci.ub	0.511	0.106		0.195	0.237		0.343	0.458	
**F**									
tau^2	0.000	0.000		0.000	0.000		0.000	0.000	
tau	0.000	0.000		0.000	0.000		0.000	0.000	
I^2	0.000	0.000		0.000	0.000		0.000	0.000	
H^2	1.000	1.000		1.000	1.000		1.000	1.000	
Q	2.023	0.039		0.983	0.458		0.601	0.065	
Qp	0.568	0.981		0.805	0.795		0.896	0.968	
**Estimate**	**-0.167**	**-0.134**		**0.047**	**-0.069**		**0.107**	**0.038**	
SE	0.206	0.259		0.205	0.259		0.205	0.259	
Z	-0.811	-0.517		0.230	-0.267		0.520	0.146	
**P**	0.418	0.605		0.818	0.789		0.603	0.884	
ci.lb	-0.570	-0.642		-0.355	-0.576		-0.295	-0.470	
ci.ub	0.237	0.374		0.450	0.438		0.508	0.545	
**Moderated analysis: SEX**									
	**N>0**			**R>0**			**R>N**		
	**0m**	**6m**	**Both**	**0m**	**6m**	**Both**	**0m**	**6m**	**Both**
**QE**	4.990	0.475	7.460	2.271	1.382	3.801	5.212	1.027	6.468
**QEdf**	6.000	4.000	12.000	6.000	4.000	12.000	6.000	4.000	12.000
**QEp**	0.545	0.976	0.826	0.893	0.847	0.987	0.517	0.906	0.891
**QM**	0.620	0.576	0.015	0.864	0.335	1.194	1.093	0.069	0.948
**QMdf**	1.000	1.000	1.000	1.000	1.000	1.000	1.000	1.000	1.000
**QMp**	0.431	0.448	0.903	0.353	0.563	0.275	0.296	0.793	0.330

Temporal RH, HbO									
	N>0			R>0			R>N		
	**0m**	**6m**	**Both**	**0m**	**6m**	**Both**	**0m**	**6m**	**Both**
**M+F**									
tau^2	0.000	0.000	0.000	0.000	0.000	0.000	0.000	0.000	0.000
tau	0.000	0.000	0.000	0.000	0.000	0.000	0.000	0.000	0.000
I^2	0.000	0.000	0.000	0.000	0.000	0.000	0.000	0.000	0.000
H^2	1.000	1.000	1.000	1.000	1.000	1.000	1.000	1.000	1.000
Q	1.268	0.357	1.689	2.838	0.509	3.956	1.950	0.676	2.671
Qp	0.737	0.837	0.946	0.417	0.775	0.683	0.583	0.713	0.849
**Estimate**	**0.297**	**0.237**	**0.274**	**0.499**	**0.312**	**0.425**	**0.150**	**0.101**	**0.131**
SE	0.149	0.185	0.116	0.151	0.186	0.117	0.148	0.185	0.116
**Z**	1.999	1.281	2.360	3.309	1.681	3.629	1.013	0.544	1.131
**P**	0.046	0.200	0.018	0.001	0.093	0.000	0.311	0.586	0.258
**ci.lb**	0.006	-0.126	0.046	0.204	-0.052	0.195	-0.140	-0.262	-0.096
**ci.ub**	0.588	0.600	0.501	0.795	0.677	0.654	0.441	0.463	0.357
**M**									
tau^2	0.000	0.026		0.000	0.000		0.000	0.000	
tau	0.000	0.161		0.000	0.000		0.000	0.000	
I^2	0.000	10.868		0.000	0.000		0.000	0.000	
H^2	1.000	1.122		1.000	1.000		1.000	1.000	
Q	0.294	2.390		1.236	0.018		1.672	1.024	
Qp	0.961	0.303		0.744	0.991		0.643	0.599	
**Estimate**	**0.424**	**0.042**		**0.396**	**0.287**		**0.118**	**0.274**	
SE	0.219	0.282		0.220	0.265		0.219	0.265	
Z	1.933	0.151		1.801	1.083		0.537	1.032	
**P**	0.053	0.880		0.072	0.279		0.591	0.302	
ci.lb	-0.006	-0.510		-0.035	-0.232		-0.312	-0.246	
ci.ub	0.854	0.595		0.826	0.806		0.547	0.794	
**F**									
tau^2	0.000	0.000		0.000	0.000		0.000	0.000	
tau	0.000	0.000		0.000	0.000		0.000	0.000	
I^2	0.000	0.000		0.000	0.000		0.000	0.000	
H^2	1.000	1.000		1.000	1.000		1.000	1.000	
Q	0.233	0.277		1.424	0.222		1.874	0.212	
Qp	0.972	0.871		0.700	0.895		0.599	0.900	
**Estimate**	**0.160**	**0.311**		**0.278**	**0.450**		**0.113**	**0.214**	
SE	0.205	0.260		0.206	0.262		0.206	0.260	
Z	0.781	1.196		1.348	1.715		0.551	0.824	
**P**	0.435	0.232		0.178	0.086		0.581	0.410	
ci.lb	-0.242	-0.199		-0.126	-0.064		-0.290	-0.295	
ci.ub	0.563	0.822		0.682	0.963		0.516	0.724	
**Moderated analysis: SEX**									
	N>0			R>0			R>N		
	**0m**	**6m**	**Both**	**0m**	**6m**	**Both**	**0m**	**6m**	**Both**
**QE**	0.527	2.667	4.669	2.660	0.240	3.265	3.546	1.236	5.081
**QEdf**	6.000	4.000	12.000	6.000	4.000	12.000	6.000	4.000	12.000
**QEp**	0.997	0.615	0.968	0.850	0.993	0.993	0.738	0.872	0.955
**QM**	0.770	0.548	0.043	0.153	0.191	0.001	0.000	0.026	0.015
**QMdf**	1.000	1.000	1.000	1.000	1.000	1.000	1.000	1.000	1.000
**QMp**	0.380	0.459	0.836	0.696	0.662	0.973	0.988	0.872	0.901

Temporal RH, HbR									
	N>0			R>0			R>N		
	0m	6m	Both	0m	6m	Both	0m	6m	Both
**M+F**									
tau^2	0.000	0.040	0.000	0.000	0.000	0.000	0.000	0.000	0.000
tau	0.000	0.201	0.000	0.000	0.000	0.000	0.000	0.000	0.000
I^2	0.000	27.570	0.000	0.000	0.000	0.000	0.000	0.000	0.000
H^2	1.000	1.381	1.000	1.000	1.000	1.000	1.000	1.000	1.000
Q	0.109	2.904	3.194	2.173	0.543	2.940	1.277	1.952	3.417
Qp	0.991	0.234	0.784	0.537	0.762	0.816	0.734	0.377	0.755
**Estimate**	-0.177	-0.264	-0.216	-0.277	-0.165	-0.233	-0.089	0.014	-0.049
SE	0.148	0.220	0.116	0.149	0.185	0.116	0.148	0.185	0.116
**Z**	-1.193	-1.197	-1.861	-1.863	-0.893	-2.010	-0.601	0.075	-0.423
**P**	0.233	0.231	0.063	0.063	0.372	0.044	0.548	0.941	0.672
**ci.lb**	-0.467	-0.695	-0.443	-0.569	-0.527	-0.460	-0.379	-0.349	-0.276
**ci.ub**	0.113	0.168	0.011	0.014	0.197	-0.006	0.201	0.377	0.178
**M**									
tau^2	0.000	0.000		0.000	0.022		0.000	0.031	
tau	0.000	0.000		0.000	0.150		0.000	0.176	
I^2	0.000	0.000		0.000	9.194		0.000	12.547	
H^2	1.000	1.000		1.000	1.101		1.000	1.143	
Q	1.542	1.774		0.827	2.471		0.694	2.383	
Qp	0.672	0.412		0.843	0.291		0.875	0.304	
**Estimate**	-0.118	-0.416		-0.389	-0.283		-0.314	0.093	
SE	0.217	0.268		0.218	0.284		0.218	0.286	
Z	-0.543	-1.551		-1.780	-0.997		-1.438	0.326	
**P**	0.587	0.121		0.075	0.319		0.150	0.744	
ci.lb	-0.544	-0.942		-0.817	-0.839		-0.741	-0.467	
ci.ub	0.308	0.110		0.039	0.273		0.114	0.653	
**F**									
tau^2	0.000	0.000		0.000	0.000		0.000	0.000	
tau	0.000	0.000		0.000	0.000		0.000	0.000	
I^2	0.000	0.000		0.000	0.000		0.000	0.000	
H^2	1.000	1.000		1.000	1.000		1.000	1.000	
Q	1.369	1.501		1.750	1.256		1.060	0.195	
Qp	0.713	0.472		0.626	0.534		0.787	0.907	
**Estimate**	-0.263	-0.117		-0.251	-0.128		-0.045	-0.024	
SE	0.206	0.260		0.206	0.260		0.205	0.259	
Z	-1.277	-0.451		-1.218	-0.491		-0.219	-0.092	
**P**	0.202	0.652		0.223	0.624		0.827	0.926	
ci.lb	-0.666	-0.627		-0.656	-0.637		-0.446	-0.531	
ci.ub	0.141	0.393		0.153	0.382		0.357	0.483	
**Moderated analysis: SEX**									
	N>0			R>0			R>N		
	0m	6m	Both	0m	6m	Both	0m	6m	Both
**QE**	2.912	3.275	7.124	2.577	3.727	6.549	1.754	2.579	5.778
**QEdf**	6.000	4.000	12.000	6.000	4.000	12.000	6.000	4.000	12.000
**QEp**	0.820	0.513	0.849	0.860	0.444	0.886	0.941	0.631	0.927
**QM**	0.234	0.639	0.016	0.209	0.157	0.360	0.807	0.111	0.228
**QMdf**	1.000	1.000	1.000	1.000	1.000	1.000	1.000	1.000	1.000
**QMp**	0.628	0.424	0.900	0.647	0.692	0.549	0.369	0.739	0.633

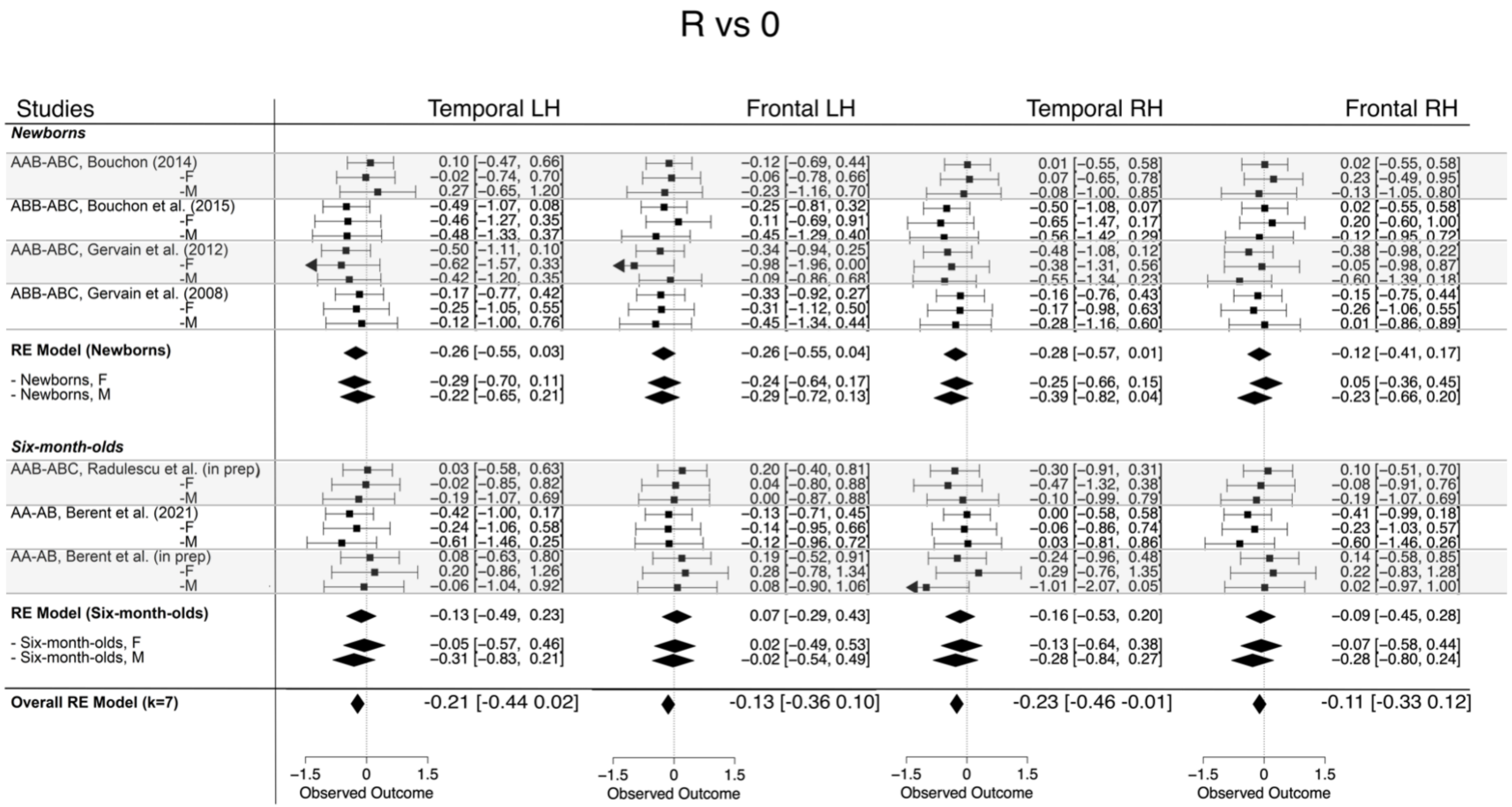

Figure S1: Forest plots of the effect sizes and corresponding confidence intervals obtained for responses elicited by repetition-based sequences compared to baseline, for the HbR component. The corresponding HbO figure is reported in the main text as Figure 3.

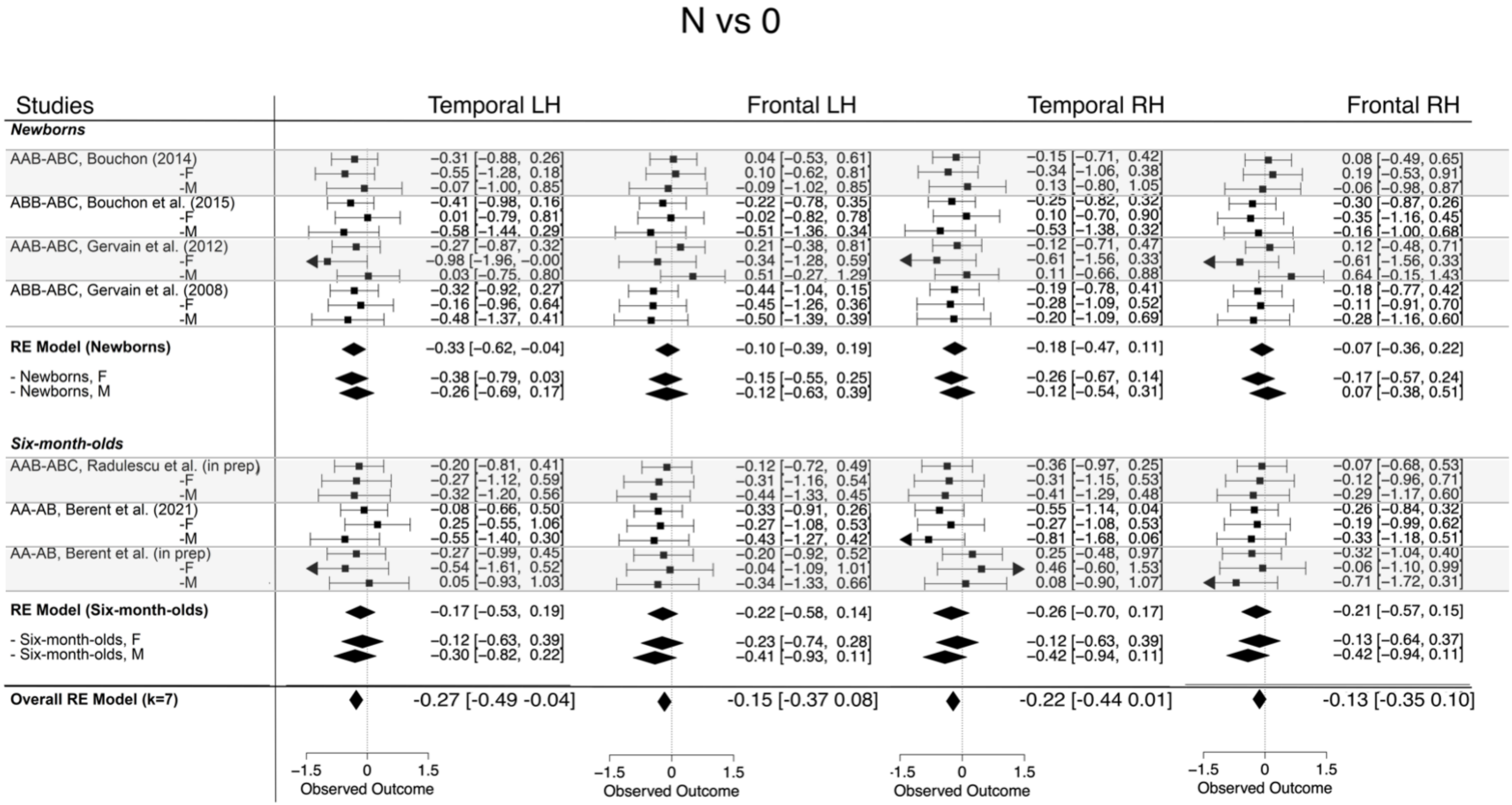

Figure S2: Forest plots of effect sizes and corresponding confidence intervals obtained for responses elicited by non-repetition-based sequences compared to baseline, obtained with the HbR component. Corresponding HbO figure is Figure 4 in the main text.

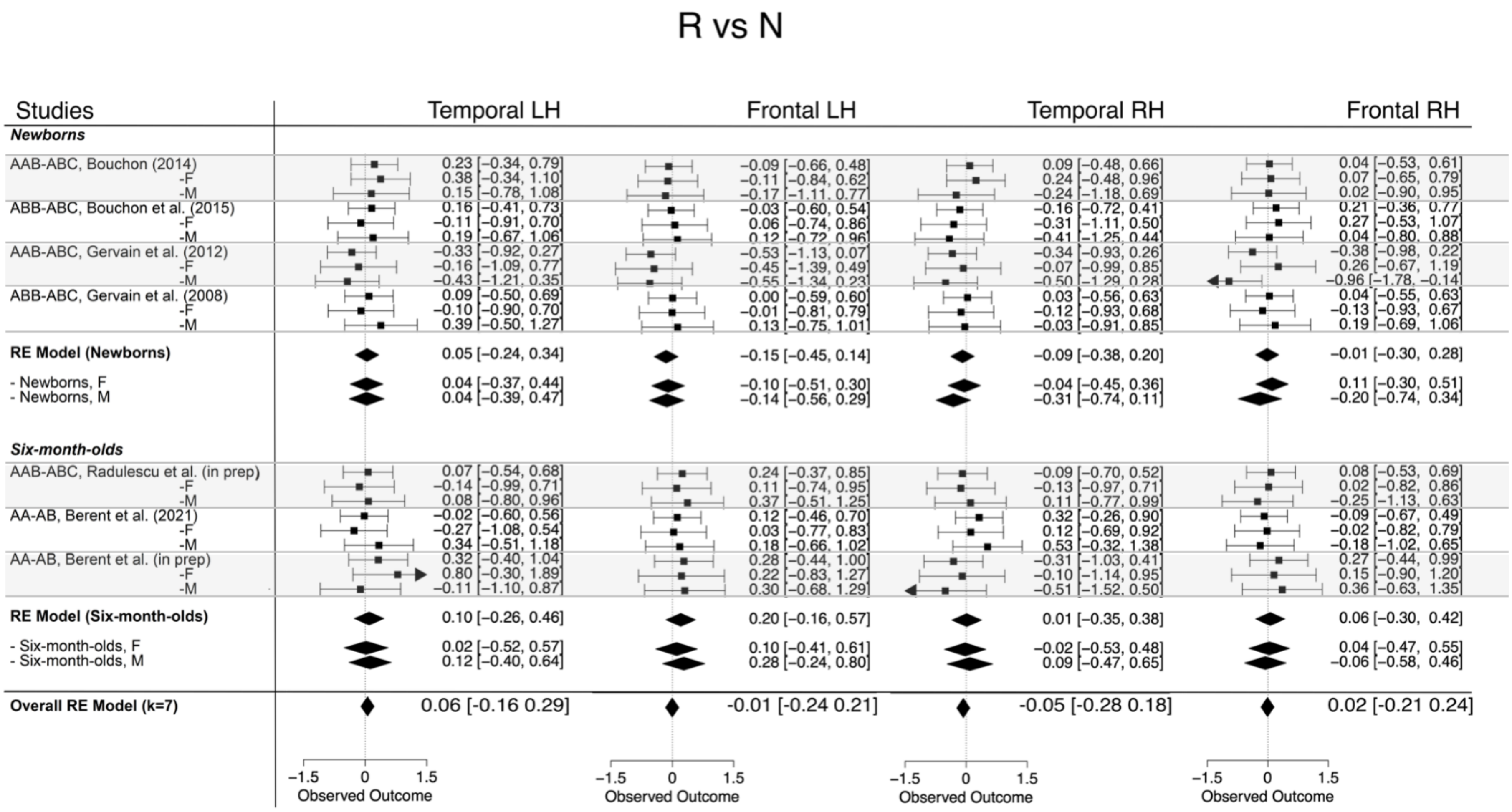

Figure S3: Forest plots of effect sizes and corresponding confidence intervals obtained for responses elicited by repetition-based sequences compared to non-repetition-based sequences, for the HbR component. Corresponding HbO figure is reported in the main text as Figure 5.

2. **Linear mixed effects models**

2.1 Anatomically derived regions

2.1.1. Model comparison results

As a result of the model comparison procedure, the following models were selected for each subset of data as the ones yielding the lowest Akaike Information Criterion (AIC) (in **bold**: models with significant effects at $p < 0.05$):

	HbO	HbR
R vs 0, Newborns	**Model 1 (1 FE: ROI)**	Model 2 (1 FE: Hemisphere)
R vs 0, Six-month-olds	Model 2 (1 FE: Hemisphere)	Model 1 (1 FE: ROI)
N vs 0, Newborns	Model 1 (1 FE: ROI)	Model 7 (3 FE: ROI x Hemisphere)
N vs 0, Six-month-olds	**Model 1 (1 FE: ROI)**	Model 1 (1 FE: ROI)
R vs N, Newborns	Model 1 (1 FE: ROI)	Model 3 (1 FE: Sex)
R vs N, Six-month-olds	Model 2 (1 FE: Hemisphere)	Model 1 (1 FE: ROI)

In particular, the following models were compared:

- **Newborns**

**Fixed effects**	**R vs 0 (HbO)**	**N vs 0 (HbO)**	**R vs N (HbO)**	**R vs 0 (HbR)**	**N vs 0 (HbR)**	**R vs N (HbR)**
ROI	**620.69**	**768.14**	**406.08**	576.09	639.72	434.30
Hemisphere	631.50	772.00	407.66	**575.67**	639.22	433.66
Sex	631.62	770.76	408.70	576.32	639.27	**430.69**
ROI + Hemisphere	625.47	773.59	414.24	581.75	642.91	440.11
ROI + Sex	625.67	772.34	413.28	580.41	643.02	437.16
Sex + Hemisphere	636.46	776.20	412.86	582.01	642.51	436.51
ROI*Hemisphere	630.01	777.46	419.32	584.13	**634.47**	443.27
ROI*Sex	629.42	775.75	417.65	582.58	647.53	442.24
Sex*Hemisphere	640.72	778.59	417.82	585.57	646.65	439.80
Hemisphere*Gender + ROI	634.75	780.09	424.40	589.63	650.38	446.26
ROI*Sex + Hemisphere	634.21	781.20	423.81	588.24	650.71	448.05
ROI*Hemisphere + Gender	635.00	781.65	424.52	588.44	647.69	446.10
ROI*Hemisphere*Gender	645.79	789.75	436.54	597.15	659.51	458.14

- **Six-month-olds**

**Fixed effects**	**R vs 0 (HbO)**	**N vs 0 (HbO)**	**R vs N (HbO)**	**R vs 0 (HbR)**	**N vs 0 (HbR)**	**R vs N (HbR)**
ROI	448.90	**519.30**	342.14	**496.59**	**523.34**	**343.37**
Hemisphere	**447.41**	521.99	**340.36**	500.37	524.65	344.66
Sex	449.84	52.48	340.57	499.95	524.85	343.93
ROI + Hemisphere	453.64	526.75	345.70	503.88	528.92	348.72

ROI + Sex	452.07	524.24	345.90	500.49	528.12	347.97
Sex + Hemisphere	452.57	523.96	344.11	501.26	528.44	349.27
ROI*Hemisphere	456.68	529.36	349.55	506.17	532.26	352.21
ROI*Sex	455.51	527.29	349.70	503.48	531.38	351.19
Sex*Hemisphere	455.76	526.43	346.42	503.79	531.80	353.24
Hemisphere*Gender + ROI	459.98	531.18	351.77	507.35	536.06	357.31
ROI*Sex + Hemisphere	460.26	531.75	353.28	507.81	535.96	356.55
ROI*Hemisphere + Gender	459.83	531.29	353.29	507.13	536.05	356.81
ROI*Hemisphere*Gender	468.40	538.72	361.95	513.83	544.21	363.93

2.1.2. Models' summary

A summary of the models yielding significant effects is reported below.

- R vs 0, Newborns (HbO)

d ~ ROI + (1 | SubjectID) + (1 | StudyID) + (1 | Lab)

	Sum Sq	Mean Sq	NumDF	DenDF	F value	Pr(>F)
ROI	4.400707	4.400707	1	249.2148	13.18262	0.0003429

Post-hoc comparison:

ROI	emmean	SE	df	lower CL	upper.CL
Frontal	0.00949	0.0915	5.79	-0.21628	0.235
Temporal	0.23315	0.0916	5.86	0.00764	0.459

Contrast	Estimate	SE	df	t	p
Frontal - Temporal	-0.224	0.0617	265	-3.623	0.0003

- N vs 0, Six-month-olds (HbO)

d ~ ROI + (1 | SubjectID) + (1 | StudyID) [1]

	Sum Sq	Mean Sq	NumDF	DenDF	F value	Pr(>F)
ROI	7.108627	7.108627	1	134.9571	4.90177	0.0285078

[1] The random intercept for Lab was removed, since all studies within this age group were carried out in the same laboratory.

Post-hoc comparison:

ROI	emmean	SE	df	lower CL	upper.CL
Frontal	0.141	0.166	3.42	-0.3520	0.634
Temporal	0.515	0.171	3.79	0.0292	1.001

Contrast	Estimate	SE	df	t	p
Frontal - Temporal	-0.374	0.17	156	-2.20	0.0290

2.1.3 Across-ages models

Linear mixed-effects model presented in the manuscript were carried out on newborns and six-month-olds separately. Below are reported linear mixed-effects models seeking to test the same effects (Sex, ROI, Hemisphere) across the two age groups, thus including Age as a categorical factors. Model comparison was carried out as described above.

- R vs 0

HbO. The best fitting model included the fixed effect of ROI, which was statistically significant (F= 9.85, $p < 0.01$), carried by a greater activation in the Temporal ROI compared to the Frontal ROI (estimate: -0.15, SE= 0.0482, $p < 0.01$).

HbR. The best fitting model included the fixed effect of Sex, but this effect was not statistically significant.

- N vs 0

HbO. The best fitting model included the fixed effect of Age, which was statistically significant (F=4.66, $p < 0.05$). The post-hoc comparison highlighted a greater activation in 6-month-olds than in newborns, but the difference did not reach statistical significance.

HbR. The best fitting model included the fixed effect of Age, which was statistically significant (F= 5.36, $p < 0.05$). Again, the contrast highlighted a greater activation in 6-month-olds, but this difference did not reach statistical significance in the post-hoc comparison.

- R vs N

HbO. The best fitting model included the fixed effect of Age, but it was not statistically significant.

HbR. The best fitting model included the interaction between ROI and Hemisphere, but there were no statistically significant main effects or interaction in the model.

2.1.4 Boxplots of effects derived from HbR

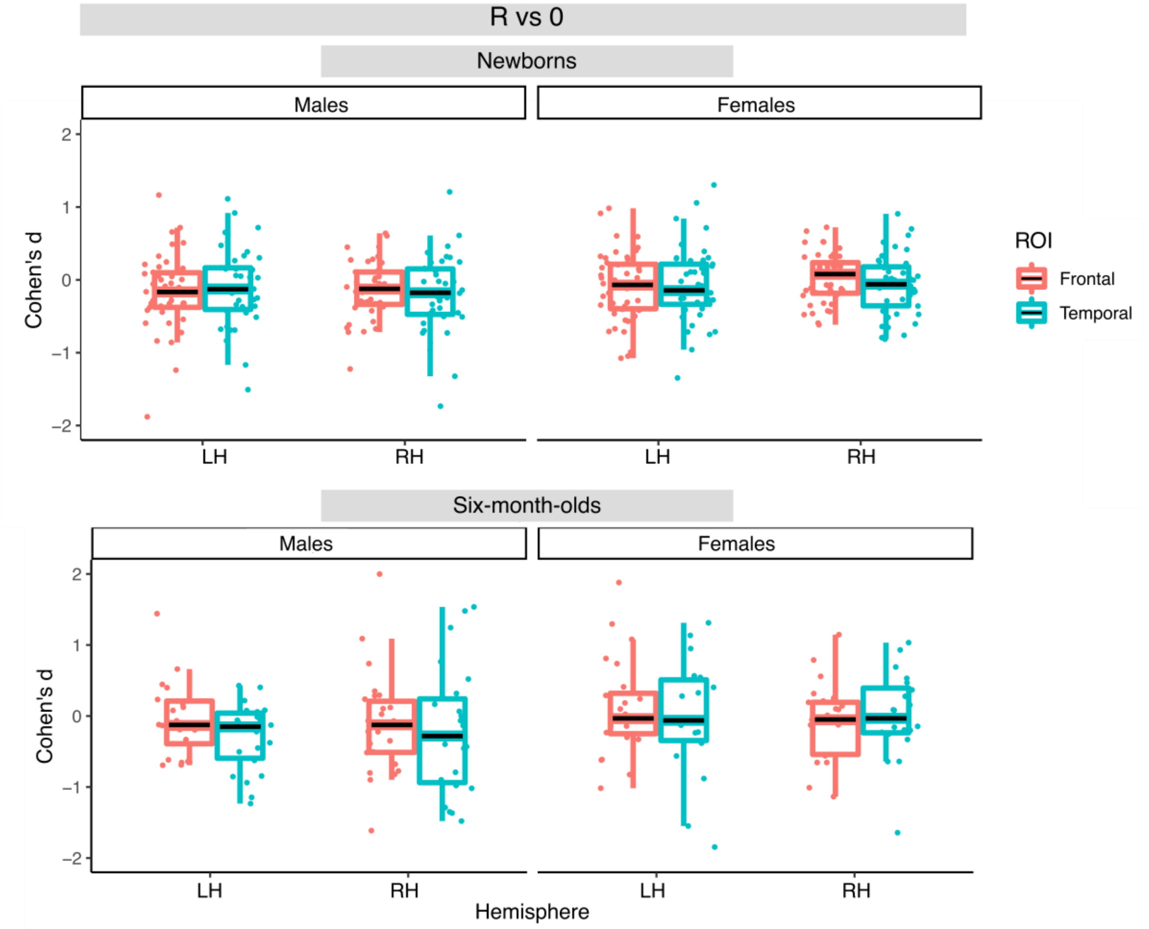

Figure S4: Box plots of infant-level effect sizes as a function of age, anatomically defined ROIs and hemisphere for responses elicited by repetition-based sequences compared to baseline, obtained from the HbR timetraces. Corresponding HbO figure in the main text is Figure 7. Boxplots display the median value of the distribution, its first and third quartiles (hinges) and whiskers extend to 1.5 times the interquartile range from each hinge.

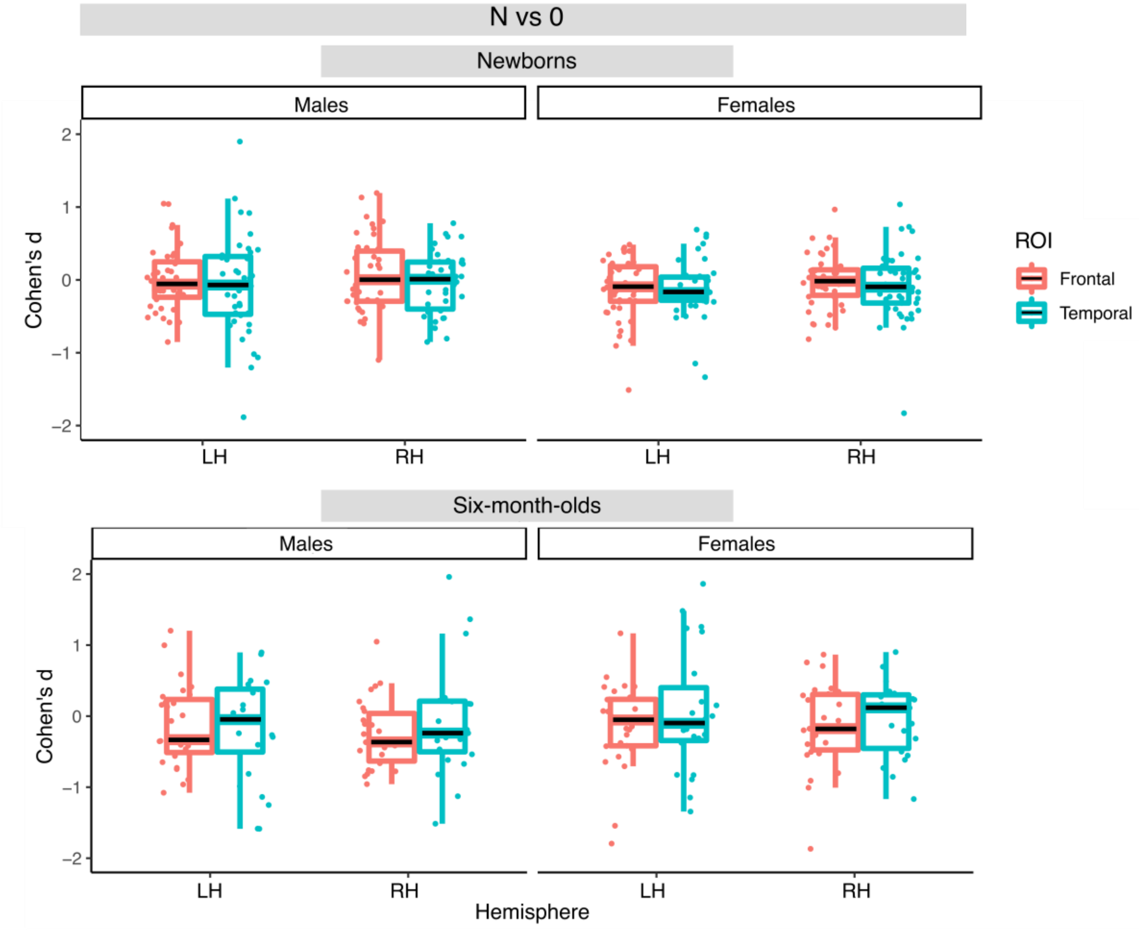

Figure S5: Box plots of infant-level effect sizes as a function of age, anatomically defined ROIs and hemisphere for responses elicited by non-repetition-based sequences compared to baseline, obtained from the HbR timetraces. Corresponding HbO figure in the main text is Figure 8.

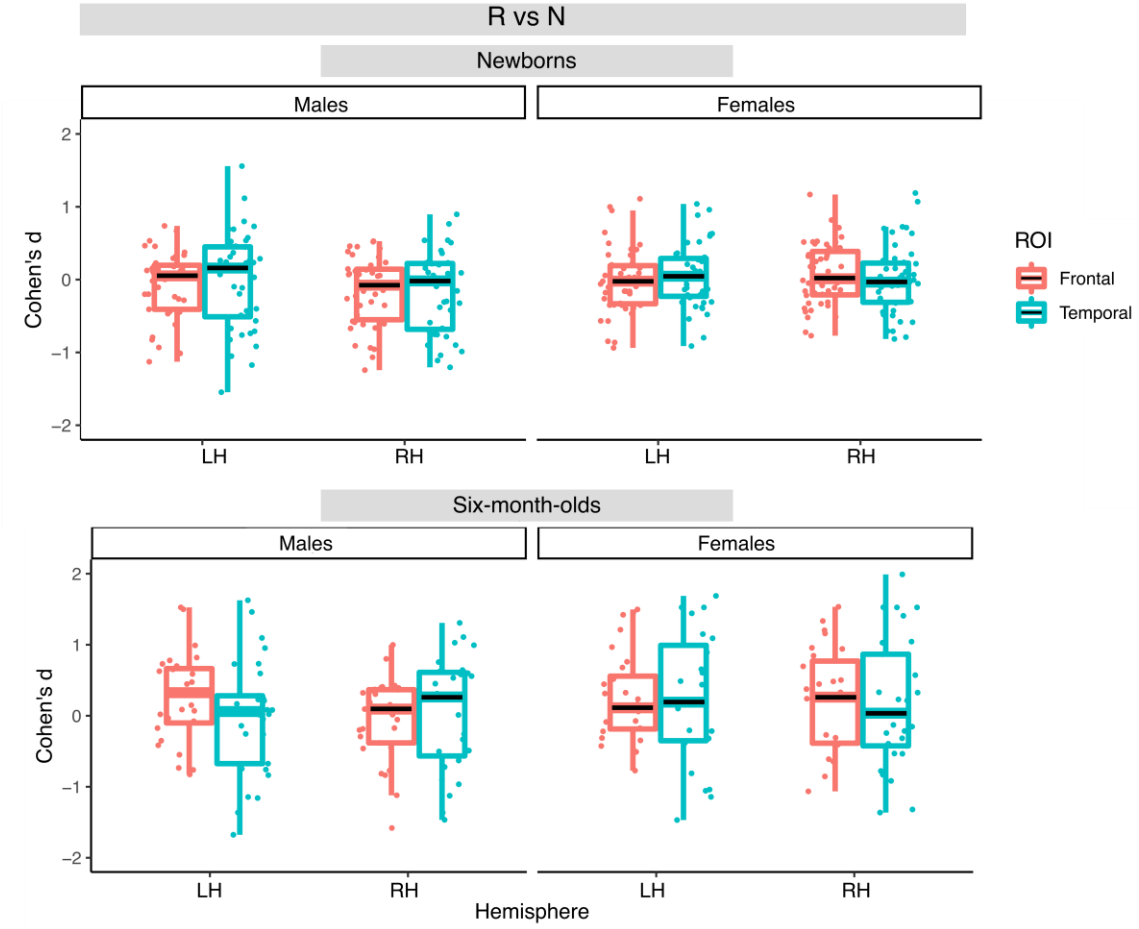

Figure S6: Box plots of infant-level effect sizes as a function of age, anatomically defined ROIs and hemisphere for responses elicited by repetition-based sequences compared to non-repetition-based sequences. Corresponding HbO figure in the main text is Figure 9.

2.1.5 Distributions of effect sizes across ages

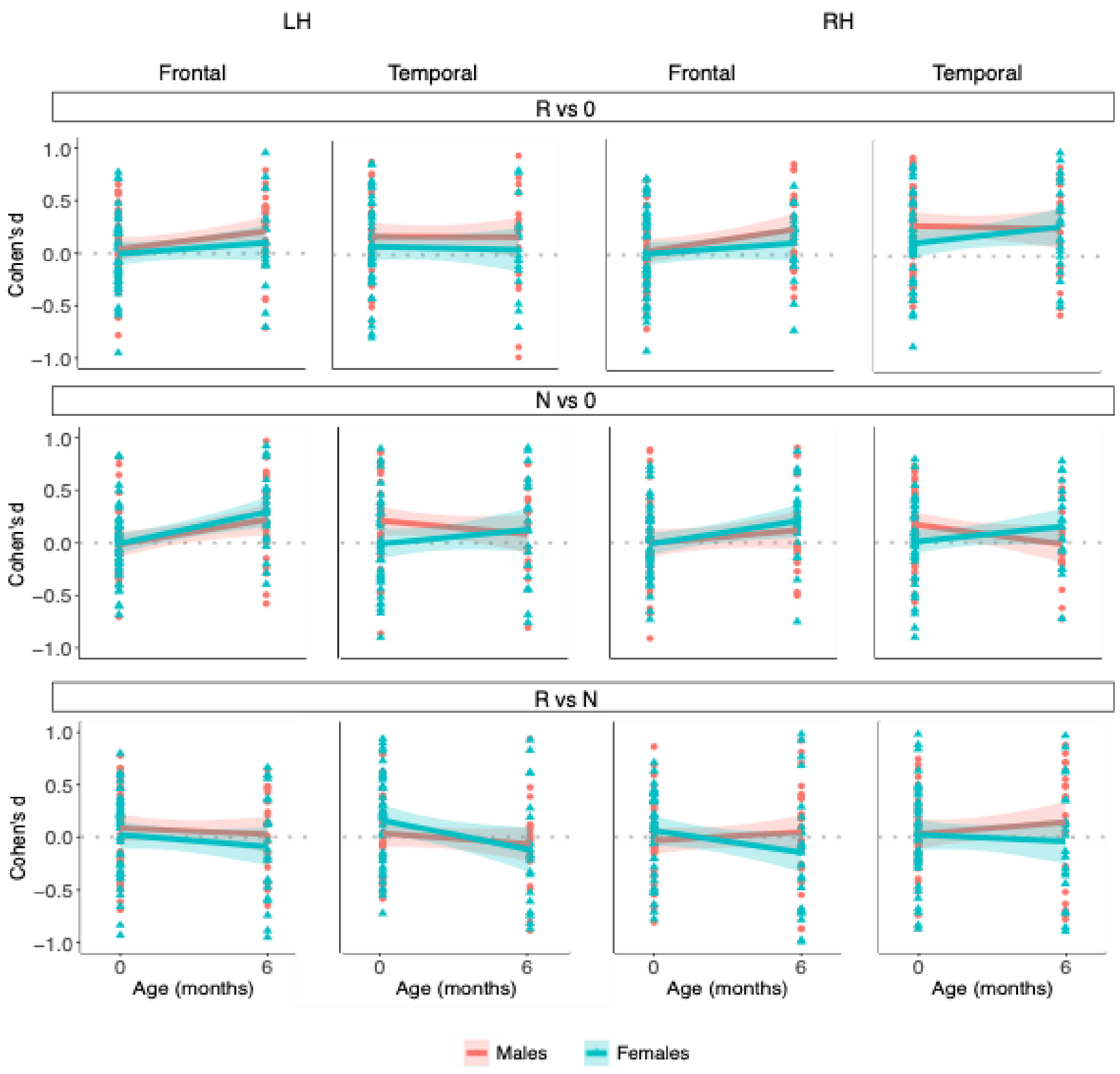

Figure S7: Effect sizes against age, split by sex, hemisphere and anatomically defined regions of interest

2.2 Functionally derived regions of interest

2.2.1 Model comparison results

As a result of the model comparison procedure, the following models were selected for each subset of data as the ones yielding the lowest Akaike Information Criterion (AIC) (in **bold**: models with significant effects at $p < 0.05$):

	HbO	HbR
R vs 0, Newborns	1 FE: Hemisphere	1 FE: Hemisphere
R vs 0, Six-month-olds	1 FE: Sex	1 FE: Sex
N vs 0, Newborns	1 FE: Sex	1 FE: Sex
N vs 0, Six-month-olds	1 FE: Sex	1 FE: Hemisphere
R vs N, Newborns	1 FE: Sex	**3 FE: Sex, Hemisphere + Sex*Hemisphere**
R vs N, Six-month-olds	**1 FE: Hemisphere**	1 FE: Sex

In particular, the following models were compared:

- **Newborns**

Fixed effects	R vs 0 (HbO)	N vs 0 (HbO)	R vs N (HbO)	R vs 0 (HbR)	N vs 0 (HbR)	R vs N (HbR)
Hemisphere	**215.57**	233.71	142.01	**339.10**	215.79	112.47
Sex	216.91	**232.42**	**141.80**	342.63	**215.59**	110.57
Hemisphere + Sex	216.80	233.50	143.46	341.09	217.59	111.92
Hemisphere*Sex	218.78	233.79	145.46	343.06	218.34	**109.43**

- **Six-month-olds**

Fixed effects	R vs 0 (HbO)	N vs 0 (HbO)	R vs N (HbO)	R vs 0 (HbR)	N vs 0 (HbR)	R vs N (HbR)
Hemisphere	161.76	223.76	**120.61**	329.84	**224.90**	93.79
Sex	**161.75**	**222.55**	125.85	**326.75**	227.47	**93.06**
Hemisphere + Sex	163.75	224.49	121.79	327.36	226.72	94.89
Hemisphere*Sex	165.72	226.34	123.49	328.99	225.69	96.76

2.2.2 Models' summary

A summary of the models yielding significant effects is reported below.

- R v N, Six-month-olds (HbO)

d ~ Hemisphere + (1 | SubjectID) + (1 | StudyID)

	Sum Sq	Mean Sq	NumDF	DenDF	F value	Pr(>F)
Hemisphere	0.65571	0.65571	1	46.14	6.419	**0.01475**

Post-hoc comparison

Hemisphere	emmean	SE	df	lower CL	upper.CL
LH	-0.0656	0.0773	7.14	-0.2477	0.117
RH	0.0994	0.0764	6.79	-0.0825	0.281

Contrast	Estimate	SE	df	t	p
LH-RH	-0.165	0.0659	51.4	-2.503	0.0155

- R vs N, Newborns (HbR)

d ~ Hemisphere*Sex + (1 | SubjectID) + (1 | StudyID) + (1 | Lab)

	Sum Sq	Mean Sq	NumDF	DenDF	F value	Pr(>F)
Hemisphere	0.021281	0.021281	1	83.225	0.4588	0.50007
Sex	0.130870	0.130870	1	89.404	2.8214	0.09650
Sex: Hemisphere	0.214149	0.214149	1	83.225	4.6169	**0.03456**

Post-hoc comparison:

Sex= Males

Hemisphere	emmean	SE	df	lower CL	upper.CL
LH	-0.0750	0.0575	17.9	-0.196	4.58e-02
RH	-0.1248	0.0598	20.3	-0.250	-8.62e-05

Contrast	Estimate	SE	df	t	p
LH – RH	0.0498	0.0503	85.2	0.990	0.32

Sex= Females

Hemisphere	emmean	SE	df	lower CL	upper.CL
LH	-0.0278	0.0550	14.5	-0.145	8.99e-02
RH	0.0679	0.0558	15.1	-0.051	1.87e-01

Contrast	Estimate	SE	df	t	p
LH – RH	-0.0956	0.0467	84.4	-2.048	0.0437

2.2.3 Across-ages models

Linear mixed-effects model presented in the manuscript were carried out on newborns and six-month-olds separately. Below are reported linear mixed-effects models seeking to test the same effects (Sex, Hemisphere) across the two age groups, thus including Age as a categorical factors. Model comparison was carried out as described above.

- R vs 0

HbO. The best fitting model included the fixed effect of Hemisphere, which did not reach statistical significance.

HbR. The best fitting model included the fixed effect of Hemisphere, which did not reach statistical significance.

- N vs 0

HbO. The best fitting model included the fixed effects of Age, Sex and their interaction, but none of them was statistically significant.

HbR. The best fitting model included the fixed effects of Hemisphere, Sex and their interaction, but none of them was statistically significant.

- R vs N

HbO. The best fitting model included the fixed effect of Hemisphere, which was statistically significant ($F= 4.14$, $p <0.05$), carried by a larger activation in the right hemisphere compared to the left (contrast LH-RH, estimate -0.0748, SE= 0.0369, $p < 0.05$).

HbR. The best fitting model included the fixed effects of Hemisphere, Age, and their interaction, but none was statistically significant.

2.2.4 Boxplots of effects derived from HbR

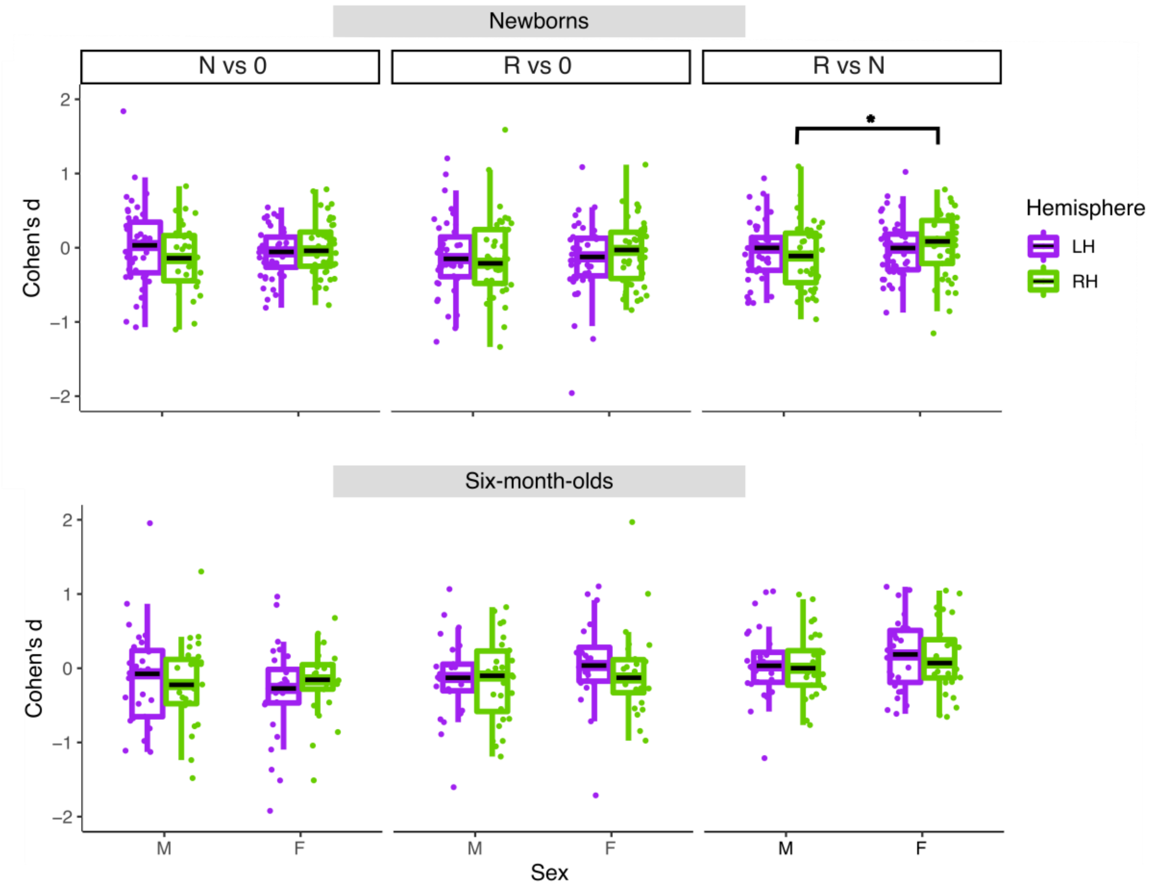

Figure S8: Box plots of infant -level effect sizes for the functionally defined ROIs in each hemisphere for the three contrasts in newborns (top panel) and 6-month-olds (bottom panel), obtained from the HbR timetraces. Corresponding HbO figure in the main text is Figure 11.
